# Nutritional and non-nutritional food components modulate phenotypic variation but not physiological trade-offs in an insect

**DOI:** 10.1038/srep29413

**Published:** 2016-07-12

**Authors:** Carlos Pascacio-Villafán, Trevor Williams, Andrea Birke, Martín Aluja

**Affiliations:** 1 Instituto de Ecología, A.C., Red de Manejo Biorracional de Plagas y Vectores, Xalapa 91070, Veracruz, Mexico

## Abstract

Our understanding of how food modulates animal phenotypes and mediate trade-offs between life-history traits has benefited greatly from the study of combinations of nutritional and non-nutritional food components, such as plant secondary metabolites. We used a fruit fly pest, *Anastrepha ludens*, to examine phenotypic variation across larval, pupal and adult stages as a function of larval food with varying nutrient balance and content of chlorogenic acid, a secondary metabolite. Larval insects that fed on carbohydrate-biased diets relative to protein exhibited longer larval and pupal developmental periods, were often heavier as pupae and resisted desiccation and starvation for longer periods in the adult stage than insects fed on highly protein-biased diets. Except for a potential conflict between pupal development time and adult desiccation and starvation resistance, we did not detect physiological trade-offs mediated by the nutritional balance in larval food. Chlorogenic acid affected *A. ludens* development in a concentration and nutrient-dependent manner. Nutrients and host plant secondary metabolites in the larval diet induced changes in *A. ludens* phenotype and could influence fruit fly ecological interactions. We provide a unique experimental and modelling approach useful in generating predictive models of life history traits in a variety of organisms.

The nutritional balance, quantity and quality of food modulate an individual’s phenotype and also mediate trade-offs between life-history traits of consumers, including insects^1^,^2^,^3^ and vertebrates^4^,^5^. Nutritional and non-nutritional food components, such as plant secondary metabolites, modify the behaviour and mediate the development and health of consumers^6^,^7^,^8^. As a result, the ecology and the evolution of animal life-history traits are influenced by food or, more specifically, by the various components present in food^9^,^10^.

The study of diet-induced phenotypic variation in life-history traits of organismal, ecological and evolutionary significance has attracted particular attention in biological and medical fields^11^,^12^. The nutritional bases of aging, starvation and desiccation resistance, and immune system function have been explored using insects as model organisms^3^,^11^,^13^. Nutritional sciences, particularly nutritional ecology and physiology, have developed much of the present theoretical understanding based on studies with insects^6^,^14^. A major challenge in these fields is to explore how the interactions among food components affect the phenotype of animals with similar and contrasting life histories^15^,^16^,^17^.

Immature development, survival, and adult emergence of flies (Diptera) are often negatively affected by high concentrations of amino acids or proteins in the larval food^2^,^18^, whereas high carbohydrate content has been associated with reduced pupal weights^19^, and improved desiccation and starvation resistance^13^. Similarly, dipteran development is often impaired by high concentrations of secondary metabolites in their food^20^, although the effects of such compounds can be modulated by the balance of protein and carbohydrate^14^,^21^,^22^.

Larvae of the Mexican fruit fly, *Anastrepha ludens* (Loew) (Diptera: Tephritidae), feed on the fruit of several wild and cultivated plant species, many of which are economically important fruit crops^23^. Frugivorous insects represent a feeding guild that remains largely unexplored in the nutritional biology of arthropods^14^,^24^. Here, we examined the hypothesis that early life nutrition has cascading effects across the larval, pupal and adult phenotypes, shaping life history of *A. ludens*. We used response surface methods^25^ to model larval survival to pupation, larval and pupal development time, pupal weight, adult emergence, and adult desiccation and starvation resistance of *A. ludens* as a function of larval diet comprising varying proportions of yeast (a protein source) and sucrose (carbohydrate) (Table 1), and varying concentrations of chlorogenic acid. Chlorogenic acid is a low molecular weight phenolic compound involved in plant defence against phytophagous insects^26^,^27^, and it is found in varying amounts in apple (*Malus* × *domestica* Borkh) cultivars, some of which are resistant to *A. ludens* attack^20^. This compound has the ability to interact with proteins to reduce their nutritional value^28^, and affect insect development and survival^29^,^30^. We further examined the relationship among larval, pupal and adult life-history traits for signs of physiological trade-offs mediated by nutrients in the larval diet, as is predicted by resource allocation theory^31^.

## Methods

### Experimental Flies

A laboratory colony of *A. ludens* started in 1998 was maintained on artificial diet in the laboratories of the Red de Manejo Biorracional de Plagas y Vectores of the Instituto de Ecología A.C., Xalapa, Mexico (RMBPV). This colony originated from pupae recovered from field-collected citrus fruit and was initially provided by the Comité Estatal de Sanidad Vegetal (DGSV-SAGARPA) in Xalapa, Veracruz^32^. At the moment of the study, the *A. ludens* colony had been laboratory-reared in the RMBPV for over 100 generations, and the last introduction of wild flies to the colony (25 females and 27 males) was made eight months before. Details on rearing of *A. ludens* have been published elsewhere^32^. In short, parental flies aged 13–16 days were held in 30 × 30 × 60 cm plexiglas cages, and provided *ad libitum* access to water and food consisting of a mixture of hydrolysed protein and refined sucrose. Flies oviposited on transparent silicon substrates. Eggs were collected from the oviposition medium and washed in 0.2% (wt/vol) sodium benzoate solution, rinsed with tap water, placed on pieces of terylene cloth on top of moistened cotton inside Petri dishes, and incubated in the dark at 30 ± 1 °C and 70 ± 5% relative humidity for four days until they hatched. On the day of hatching, neonate larvae were used in experiments.

### Experimental Diets

Experimental diets were modifications of the formula used for mass-rearing *A. ludens* at the Moscafrut sterile insect production plant at Metapa, Chiapas, Mexico^33^. Diets were oligidic, i.e., most of their ingredients were not chemically defined, with inactivated torula yeast (*Candida utilis*) as source of protein and sucrose as source of digestible carbohydrate.

#### Experiment 1

A basic diet was prepared consisting of (percentage by weight): 6.20% corn flour, 22.1% corncob fractions, 0.12% guar gum, 0.12% nipagin, 0.47% sodium benzoate, 0.50% citric acid and 70.49% water. Portions of 21.5 g (wet wt) of the basic diet were thoroughly mixed with 3.55 g (dry wt) of each of 17 yeast × sucrose mixtures (Table 1). In this way, 85 portions of diet were prepared (17 diets × 5 replicates), each weighing 25 g.

The protein, lipid, carbohydrate and energy content of yeast and sucrose was analysed at the Laboratorios de Alta Tecnología de Xalapa, S.C. (LATEX), in Xalapa, Veracruz, Mexico, following standard bromatological methods^34^. We used this information to estimate the nutrient and calorie contribution of each yeast × sucrose mixture to the diets (Table 1 and Supplementary Table S1).

#### Experiment 2

A low yeast-high sucrose and a high yeast-low sucrose diet (Table 1 and Supplementary Table S1) were prepared. We took 11 portions from each of these diets, each consisting of 100 g, and placed each of them in 0.5 L plastic containers (11 cm in diameter by 7 cm in height). Simultaneously, a 1% (wt/vol) stock solution of chlorogenic acid (purity ≥ 95%, Sigma-Aldrich Company, Toluca, Mexico) was prepared by dissolving 622 mg of chlorogenic acid in a solution of 8% ethanol (vol/vol) in water. Portions of diet in 0.5 L plastic containers were treated with 16 or 32 mg of chlorogenic acid from the stock solution and mixed by stirring with a spatula for three minutes. Control diets were treated with an identical volume of 8% ethanol solution alone. The higher concentration tested (32 mg/100 g) was within the range of chlorogenic acid concentrations present in apple cultivars that are resistant to *A. ludens* attack^20^,^35^.

### Experimental Designs

#### Experiment 1

We used a mixture experiment^36^ to evaluate the proportional effects of yeast × sucrose mixtures on larval, pupal and adult phenotypes of *A. ludens* (see *Response variables* for details on traits measured). The experiment was a two component (yeast and sucrose) mixture design, constructed using D-optimal criteria to provide the most accurate estimates of the model coefficients to satisfy a quadratic polynomial model^25^. The design was modified to include additional points for lack of fit and error estimation^25^. Overall, the experiment consisted of 17 yeast × sucrose mixtures used to prepare 17 artificial diets, each replicated five times (Table 1). The experimental design layout is presented in Supplementary Table S2.

#### Experiment 2

We used a randomized two-factor design to evaluate the effects of nutrient ratios in the larval diet, chlorogenic acid and their interactions on the development and survival of *A. ludens* (see *Response variables* section). Diet had two levels with different nutrient ratios: high yeast-low sucrose and low yeast-high sucrose (Supplementary Table S1). Chlorogenic acid was added to the diets at one of two concentrations: 16 or 32 mg/100 g of artificial diet (Supplementary Table S3). The experiment consisted of six diet × chlorogenic acid combinations, each of which was replicated three or four times (Supplementary Table S3).

### Experimental Procedure

Samples of 20 g of each diet were placed in plastic Petri dishes (5 cm diameter × 2 cm tall) without a lid, together with 25 and 20 *A. ludens* neonate larvae for *Experiment 1* and *Experiment 2*, respectively. Petri dishes with diet and larvae were placed individually inside plastic containers (7 cm diameter × 6 cm height) containing a 1 cm layer of vermiculite as a pupation substrate. Plastic containers were closed with a lid that had a 5 cm diameter hole covered with organdy cloth, and placed in a dark room at 30 ± 1 °C and 70 ± 5% RH. Petri dishes with diet and larvae were the experimental units. To pupate, larvae crawl from the diet into the vermiculite. Pupation was checked daily, beginning seven days after the start of the experiment, by removing Petri dishes with diet from the plastic containers and sifting vermiculite. Five days after the beginning of pupation, diets were also inspected for pupae. All pupae found in vermiculite and/or diet were removed and placed in Petri dishes (4 cm diameter × 1.5 cm tall) with a perforated lid to allow ventilation. Pupae were incubated at 27 ± 1 °C, 63 ± 5% RH, and photoperiod of 12: 12 (light: dark). When pupae of *Experiment 1* and *Experiment 2* reached 11 and three days old, respectively, they were weighed using an analytical balance (Sartorius CP64), and individually placed inside plastic cells (1.6 × 1.6 cm) of a compartmentalized dish, covered with a transparent acrylic lid with perforations to allow ventilation, until adult emergence and death.

### Response Variables

#### Experiment 1

The following response variables were measured: (1) survival to pupa, expressed as the proportion of individuals reaching the pupal stage in relation to total number of larvae placed in each Petri dish with diet; (2) duration of the larval stage, estimated as the mean of the difference between day of egg hatch and day of pupation; (3) mean pupal weight (mg), estimated by weighing each cohort of pupae 11 days after pupation, summing the weight of all cohorts within a single replicate and dividing it by the total number of pupae per replicate; (4) adult emergence, expressed as the proportion of adults emerged in relation to the total number of pupae recovered from each diet; (5) duration of the pupal stage, calculated as the mean of the difference of day of adult emergence minus day of pupation; (6) adult desiccation and starvation resistance, measured every 12 h (at 09.00 and 21.00 hours) from adult emergence until death, and expressed as the mean adult lifespan in hours in the absence of food or water. Individuals were considered dead when they were immobile and did not respond to the touch of a plastic probe.

#### Experiment 2

The response variables were the same as described in *Experiment 1,* but instead of survival to pupae and adult emergence, we measured immature survival and immature development time to adulthood.

### Statistical Analyses

Data for each response variable were first explored graphically to visualize the spread of the data^37^. *A priori* data exploration was followed by graphical examination of outliers and influential data points after model fitting. Response surface methods (RSM) were used to model the data^25^. In *Experiment 1*, Scheffé polynomial models from the linear to the quartic were fitted sequentially for each response variable, with yeast × sucrose mixtures as explanatory variables. Scheffé polynomial models are a form of predictive models designed specifically for mixtures accounting for the constraint that all mixtures sum to a constant^25^,^36^. In *Experiment 2*, in addition to the range of standard models, a two-factor interaction model was also fitted, with diet (high yeast–low sucrose and low yeast–high sucrose), chlorogenic acid content and the diet × chlorogenic acid content interaction as explanatory variables. Model selection was then based on: (a) lack of any aliased terms; (b) low residuals; (c) a low model *P*-value; (d) non-significant lack of fit; (e) low standard deviation; (f) high *R*^*2*^, adjusted *R*^*2*^ (*R*^*2*^_adj_) and predicted *R*^*2*^ (*R*^*2*^_pred_); (g) close agreement between R^2^_adj_ and R^2^_pred_; and (h) a low predicted residual sum of squares (PRESS) value in relation to the other models^25^. The selected model was further examined by analysis of variance (ANOVA). When possible, model simplification was performed by backward elimination of non-significant terms in the model. The relative effect of each mixture component and their interactions on the various response variables was estimated by examining the model coefficients (β). Normality and homoscedasticity were examined graphically via normal probability plots of residuals following ANOVA and by plotting the internally studentized residuals against the predicted responses^25^. Potential outlier points were identified by examination of plots of externally studentized “outlier-*t*”^38^,^39^ and Cook’s distance^40^ values. Box-Cox plots were used to identify, if required, the correct power law transformation^41^. DFFITS (a measure of influence based on the difference in fits in each predicted value) and DFBETAS (a measure of influence based on difference in model coefficients) plots were used to identify overly influential data points^42^. In *Experiment 1*, one data point was ignored for the analysis of the duration of the larval stage, pupal weight and adult emergence, and one data point for the analysis of the duration of the pupal stage. The rationale for the exclusion of these points is explained in detail in the Supplementary Information.

In *Experiment 1*, data on adult emergence were logit transformed prior to analyses to normalize and correct heteroscedasticity of residuals^25^. In *Experiment 2*, data on development time to adulthood and pupal weight were rank transformed^43^, because heteroscedasticity could not be corrected by any power law transformation.

Pearson’s correlation analyses were used to examine the association between recorded response variables from *Experiment 1*.

The software Design-Expert^®^ 8 (Stat-Ease, Inc, Minneapolis, MN) was used for experimental design construction and statistical modelling. Boxplots and Pearson’s correlation analyses were performed with the software package R^44^. Statistical significance for all tests was set at a critical value of α = 0.05.

## Results

### Experiment 1

The recorded response variables and exploratory boxplots are shown in Supplementary Table S2 and Supplementary Fig. S1, respectively. The protein, lipid, carbohydrate and calorie content of each yeast × sucrose mixture is presented in Table 1.

A summary of the ANOVA, lack of fit test and the *R*^*2*^ statistics of models fitted to all response variables, are presented in Table 2. With the exception of adult emergence, all other response variables varied significantly (*P* < 0.001) according to the composition of the yeast × sucrose mixtures tested. Lack of fit statistics for models fitted to all response variables were not significant, indicating that fitting a different model would not result in a significant reduction in the variation in residual values. Diagnostic and influence plots for each model and model discussion are presented in Supplementary Figs S3–S10.

#### Survival to pupa

**Figure 1:**
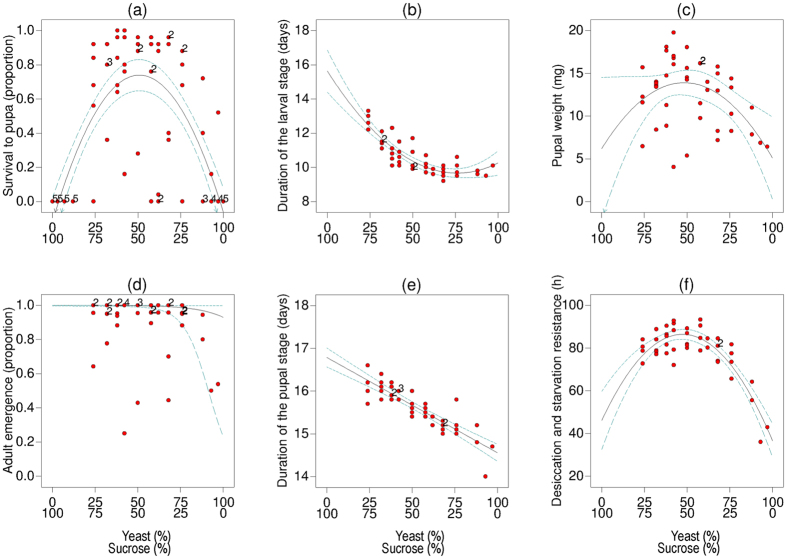
Models of best fit to data on: (**a)** survival to pupa (proportion), (**b**) duration of the larval stage (days), (**c**) pupal weight (mg), (**d**) adult emergence (proportion), (**e**) duration of the pupal stage (days), and (**f**) desiccation and starvation resistance (in hours) of *Anastrepha ludens* fed on diets with varying proportions of yeast and sucrose. The solid line represents the model fitted to the data, and dotted lines indicate the 95% confidence interval. Numbers next to circles indicate the number of observations at the same coordinate. See Table 2 for statistical information.

A quadratic model indicated that both individual yeast (β = −0.063; 95% CI: −0.20, 0.077) and sucrose (β = −0.12; 95% CI: −0.26, 0.024) components each had a negative effect on larval survival to pupae, and that the yeast × sucrose mixture (β = 3.32; 95% CI: 2.68, 3.96) was necessary to achieve survival (Table 2). However, survival was not observed in all mixtures. No larvae survived in diets with a yeast:sucrose mixture in the range of 0:100 to 12:88, or in the diet consisting of 100:0. In contrast, the proportion of larvae that pupated in diets with a range of yeast:sucrose mixtures from 24:76 to 76:24 was 1.0 or close to 1.0 (Fig. 1a). Survival to pupae in the 0:100 and 100:0 yeast:sucrose mixtures did not vary significantly (linear mixture not significant, Table 2).

#### Duration of the larval stage

The duration of the larval stage ranged from an average (±SE) of 9.2 (±0.13) days in the 68:32 yeast:sucrose mixture to 13.3 (±0.38) days in the 24:76 yeast:sucrose mixture (Fig. 1b). A quadratic model indicated that the interaction of yeast × sucrose significantly reduced larval development time (β = −10.29; 95% CI: −14.29, −6.29), whereas the relative contributions of individual sucrose (β = 15.63; 95% CI: 14.40, 16.86) and yeast (β = 10.25; 95% CI: 9.54, 10.96) components, tended to increase the duration of the larval stage (Table 2).

#### Pupal weight

The mean weight of 11 day old pupae (estimated from the weights of cohorts of pupae) ranged from 4.03 mg in the 42:58 yeast: sucrose mixture to 19.76 mg in the 42:58 yeast:sucrose mixture (Fig. 1c). A quadratic model indicated that sucrose (β = 6.17; 95% CI: −2.16, 14.50) had a stronger, but more variable positive influence on pupal weight than yeast (β = 5.09; 95% CI: 0.28, 9.90), and the yeast × sucrose interaction had the largest positive effect on pupal weight (β = 33.03; 95% CI: 5.97, 60.09) (Table 2). Pupal weights were highly variable across some of the diets evaluated, and the model explained only 17% of the observed variance (Table 2).

#### Adult emergence

The proportion of adults that emerged ranged from 0.25 in the 42:58 yeast:sucrose mixture to 1.0 in various diets among the 24:76 and 76:24 yeast:sucrose mixtures (Fig. 1d). A linear model provided the best fit to logit transformed proportions, and indicated that emergence of adult flies reared on the different diets did not vary significantly according to the mixtures tested (Table 2). However, the coefficient estimated for sucrose (β = 9.68; 95% CI: 5.51, 13.86) was larger than that of yeast (β = 2.58; 95% CI: −1.20, 6.36), suggesting a stronger positive effect of sucrose on adult emergence.

#### Duration of the pupal stage

The duration of the pupal stage ranged from an average of 14.0 days (calculated from two individuals with a duration of 14 days each) in the 93:7 yeast:sucrose mixture to 16.6 (±0.17) days in the 24:76 yeast:sucrose mixture (Fig. 1e). A linear model indicated that pupae took longer to develop to adulthood when larvae had fed on diets with high proportions of sucrose (β = 16.79; 95% CI: 16.57, 17.01), whereas they developed faster as the proportion of sucrose relative to yeast decreased (β = 14.56; 95% CI: 14.36, 14.75) (Table 2).

#### Adult desiccation and starvation resistance

The mean duration of adult survival without water and food ranged from 36.0 (±8.6) h in the 93:7 yeast:sucrose mixture to 93.3 (±2.8) h in the 58:42 yeast:sucrose mixture (Fig. 1f). A quadratic model indicated that the interaction of yeast × sucrose in the larval food significantly increased resistance to desiccation and starvation (β = 179.92; 95% CI: 135.84, 223.99), the contribution of sucrose on desiccation and starvation resistance was positive and larger (β = 46.08; 95% CI: 32.50, 59.65) than the contribution of yeast (β = 36.55; 95% CI: 28.72, 44.38) (Table 2).

#### Pearson’s correlation

**Figure 2:**
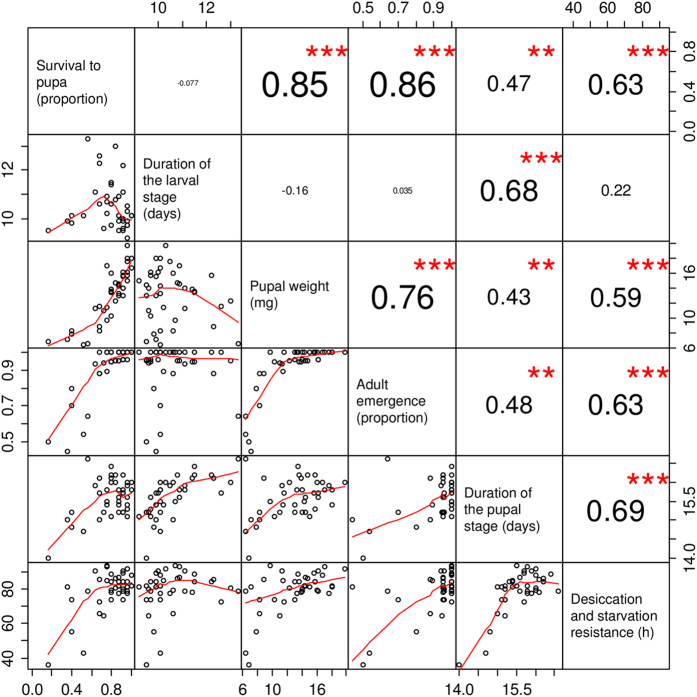
Pair-wise correlation matrix between survival to pupa, duration of the larval stage, duration of the pupal stage, pupal weight, adult emergence, and desiccation and starvation resistance of *Anastrepha ludens* reared on diets with varying proportions of yeast and sucrose. The correlation coefficients and level of significance (**P < 0.01, ***P < 0.001, absence of an asterisk indicates a non-significant correlation) are show on the right of the diagonal; the font size of the correlation coefficients is proportional to its value. The scatter-plot matrix is shown on the left side of the diagonal.

Significant correlations among the various phenotypic traits measured were all positive (Fig. 2). Adult desiccation and starvation resistance (*r* = 0.63), the duration the pupal stage (*r* = 0.47) and adult emergence (*r* = 0.86) rose significantly as the proportion of larval survival to pupae increased. The duration of the pupal stage (*r* = 0.68) was significantly correlated with the duration of the larval stage. Adult emergence (*r* = 0.76), the duration of the pupal stage (*r* = 0.43) and adult desiccation and starvation resistance (*r* = 0.59) rose significantly with pupal weight. The duration of the pupal stage (*r* = 0.48) and adult desiccation and starvation resistance (*r* = 0.63) were significantly correlated with adult emergence. Adult desiccation and starvation resistance (*r* = 0.69) rose significantly with the duration of the pupal stage.

### Experiment 2

The recorded response variables and exploratory boxplots are shown in Supplementary Table S3 and Supplementary Fig. S2, respectively.

A summary of the ANOVA, lack of fit tests and the *R*^*2*^ statistics of models fitted to all response variables are given in Table 3. Models fitted to results on duration of the larval stage and development time to adulthood were significant (*P* < 0.05). Models fitted to pupal duration, pupal weight and desiccation and starvation resistance were not significant (*P* > 0.05). Lack of fit test statistics were not significant for any of the models. Diagnostic and influence plots for each model and model discussion are presented in Supplementary Figs S11 and S12.

#### Duration of the larval stage

**Figure 3:**
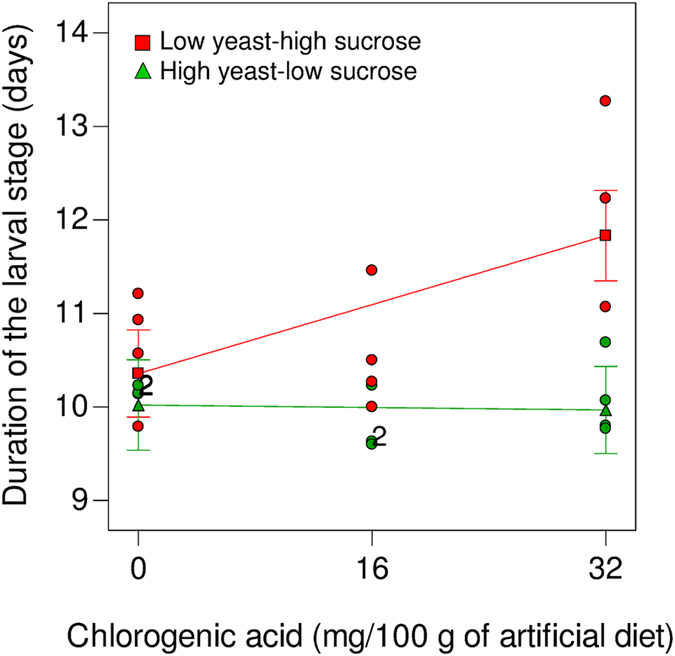
The duration of the larval stage (days) of *Anastrepha ludens* fed on low yeast-high sucrose (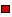) and high yeast-low sucrose (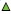) diets treated with chlorogenic acid. Circles are actual responses, and the endpoints represents mean model estimates with least significant difference bars. Numbers next to circles indicate the number of observations at the same coordinate. See Table 3 for statistical information.

Larval development time increased as the concentration of chlorogenic acid rose in the low yeast-high sucrose diet, but not in the high yeast-low sucrose diet (Fig. 3). A two-factor interaction model (Table 3) revealed a significant effect of the diet yeast:sucrose ratio (β = −0.55, 95% CI: −0.85, −0.25) and diet × chlorogenic acid interaction (β = −0.38, 95% CI: −0.75, −0.011). Larvae took longer to complete development when fed on low yeast-high sucrose diet treated with 32 mg of chlorogenic acid, compared to larvae fed on low yeast-high sucrose diets without chlorogenic acid or on high yeast-low sucrose diet with or without chlorogenic acid (Fig. 3).

#### Duration of the pupal stage

No significant effects of the diet yeast:sucrose ratio, chlorogenic acid content or their interaction were detected after backward elimination of non-significant terms (*P* ≥ 0.0673) from a two-factor interaction model. The overall mean of 15.03 ± 0.12 days provided the best description of the data (Table 3, Supplementary Fig. S2).

#### Pupal weight

The lowest mean weights of 11 day old pupae were observed in the low yeast-high sucrose content diet treated with 32 mg of chlorogenic acid (Supplementary Fig. S2). However, there was no significant effect of the diet yeast:sucrose ratio or chlorogenic acid content on pupal weight after backward elimination of non-significant terms (*P* ≥ 0.1299) from a linear model. Thus, the overall mean of 18.42 ± 0.53 mg provided the better description of the data (Table 3).

#### Survival to adult

The lowest proportion of larvae that reached adulthood was 0.4, observed in both low yeast-high sucrose and high yeast-low sucrose diets with 32 mg of chlorogenic acid/100 g of diet (Supplementary Fig. S2). The highest proportion of survival was 0.6, observed in both low yeast-high sucrose, with or without chlorogenic acid, and high yeast-low sucrose diets with either chlorogenic acid treatment (Supplementary Fig. S2). No significant effect of the yeast:sucrose ratio, chlorogenic acid content, or the interaction of yeast:sucrose ratio × chlorogenic content, was detected in survival to adulthood by any model from the linear to the cubic (*P* ≥ 0.4152). A null model indicated that the overall mean (average proportion ± SE) of 0.53 ± 0.014 provided the best description of survival to adulthood (Table 3).

#### Development time to adulthood

Development time from neonate larvae to adulthood was significantly affected by the yeast:sucrose ratio in the diet, but not by chlorogenic acid content or the yeast:sucrose ratio in the diet × chlorogenic acid interaction, as indicated by a reduced linear model (Table 3). Insects reached the adult stage faster when fed on diets with high yeast-low sucrose regardless of the chlorogenic acid treatment (Supplementary Fig. S2).

#### Adult desiccation and starvation resistance

Individuals fed as larvae on the low yeast-high sucrose diet treated with 32 mg of chlorogenic acid lived for shorter periods as adults when subjected to desiccation and starvation (Supplementary Fig. S2). However no significant effect of the yeast:sucrose ratio, chlorogenic acid content or diet × chlorogenic acid interaction was detected, as indicated by a mean model fitted after backward elimination of non-significant terms (*P* ≥ 0.0971) from a quadratic model (Table 3). The mean adult survival time without food was 85.3 ± 2.01 h.

## Discussion

Our study provides clear support to the hypothesis that early life nutrition generates cascading effects across the immature and adult phenotypes that shaped life history variables of the devastating fruit fly pest *A. ludens*. In *Experiment 1*, changes in yeast and sucrose proportions resulted in larval diets that varied in protein, lipid and carbohydrate content (Table 1, and Supplementary Table S1). As such, the availability of nutrients in the larval food resulted in phenotypic variation across the larval, pupal and adult stages of *A. ludens*. The quadratic responses observed for survival to pupa, the duration of the larval stage, pupal weight, and desiccation and starvation resistance (Fig. 1), resembled the inverted U-shape and J-shaped dose response curves from the nutritional pharmecology principle of hormesis and Bertrand’s rule^22^. This suggests that an extreme nutritional imbalance in food (protein and carbohydrate in our study), was deleterious, i.e., at extreme concentrations nutritious food components are likely to be toxic. *Anastrepha ludens* larvae appeared to be more sensitive to high carbohydrate:protein ratios in their diet than to low carbohydrate:protein ratios (Fig. 1a, Table 1). Protein consumption on diets containing 88–97% yeast relative to sucrose was tolerated by some individuals, albeit at a high cost for larval survival and adult resistance to desiccation and starvation (Fig. 1a,f).

Our results did not provide strong evidence to support the view of physiological trade-offs mediated by nutrients in the larval diet, as indicated by the lack of significant correlations and only positive associations in the correlation analyses (Fig. 2). The results suggested a trade-off in desiccation and starvation resistance and the duration of the pupal stage, that were more strongly correlated with each other than with any other measured trait. Pupae with longer developmental periods tended to exhibit higher weights and were able to resist desiccation and starvation as adults for longer periods than flies that emerged earlier from pupae with lighter weights. Rapid development to adulthood may have diverted bodily resources required to deal with water and food limitation in the adult stage, reflecting a conflicting use of resources between the rate of development to adulthood and desiccation and starvation resistance. Indeed, phenotypic variation in life history traits may reflect differential resource allocation to competing life functions^3^,^31^. To resist starvation, adult *Drosophila* flies make use of lipid reserves stored after use as energy suppliers during metamorphosis^13^,^45^. Similarly, *A. ludens* larvae that developed on diets with an elevated proportion of sucrose, may have been able to accumulate larger lipid reserves than those that fed on lower sucrose diets. Considering the nutritional contribution of corn flour and corncob fractions in the basic diet, larvae that developed to adulthood on the lowest sucrose proportion diet had an 8% lower calorie content than diets with the highest sucrose proportion (Supplementary Table S1). Energetic reserves accumulated during larval development, likely stored as lipids, could have lasted through metamorphosis and been used to resist starvation for longer periods in the adult stage. This would need confirmation, as studies with the tephritid *Ceratitis capitata* (Wiedemann) have argued that lipid accumulation in newly emerged adults is independent of the sucrose content in the larval food^18^, and that high levels of dietary carbohydrate may in fact depress the lipogenic activity of developing larvae^19^.

Growing fast may have fitness costs and benefits. For instance, reproductive success can improve if individuals reaching adulthood faster than others in the same cohort gain additional time to find mates and reproduce^46^. In contrast, growing fast may depress immune function in some insects^3^. Our results suggest that rapid development of *A. ludens* to adulthood would be disadvantageous in situations where they will face water and food shortage or when developing on a suboptimal diet, whereas individuals growing slower could be better fitted to face water and food limitation in the adult stage (Fig. 2). The prolonged larval and pupal developmental periods observed on diets with 25% yeast could suggest that larvae prolonged their feeding period to consume enough of the nutrient present in very low concentrations (e.g., protein), while at the same time avoiding overconsumption of the abundant nutrient (sucrose) in potentially toxic quantities. Nutrient intake regulation has been reported in many animals, and foraging theory has been employed to interpret this behaviour as a strategy to maximize fitness in the face of stressful nutritional environments^21^,^47^. To determine if *A. ludens* larvae modify their feeding behaviour in response to nutrient limitations in their diet, it will be necessary to develop a technique that allows the precise quantification of specific nutrient consumption. This is a challenge for dipteran larvae in general, as feeding larvae defecate when tunnelling through the diet, and faeces mix with diet making it difficult to directly and accurately measure diet consumption.

In *Experiment 2*, we examined the effects of a natural plant phenolic compound on *A. ludens* development and survival as mediated by the nutrient content of the larval food. Chlorogenic acid affected *A. ludens* larval development time in a nutrient and concentration dependent manner (Fig. 3). One possible explanation for the extended duration of the larval stage observed in diets with low yeast-high sucrose content treated with 32 mg of chlorogenic acid, is that such diets may have been more difficult to digest than alternative diets, thus slowing feeding rate and thereby extending the duration of the larval stage. Chlorogenic acid may act as antinutritional compound due to its ability to covalently bind to proteins, thus inhibiting protein digestion^28^,^48^. The combination of reduced protein (yeast) levels and a high concentration of chlorogenic acid may have reduced the insect’s ability to assimilate protein, resulting in an extended larval development time. However, this seems unlikely as the antinutritional effect of chlorogenic acid occur in guts with a pH 8–9^48^, whereas tephritid larvae have acidic guts with a pH 3.4–6.6^49^. Acid pHs do not promote formation of covalent bonds between phenolics and other compounds^50^. Alternatively, low yeast-high sucrose diets treated with a high concentration of chlorogenic acid may have been unpalatable to larvae, thus slowing the feeding rate. In fact, chlorogenic acid is recognized as an insect antifeedant^26^. Further studies should also consider exploring host plant secondary metabolites effects on tephritid larval microbiota in terms of nutrient assimilation or compensation^51^,^52^, as many plant secondary metabolites, such as chlorogenic acid, exhibit antimicrobial activity^53^.

The responses of insects to diverse nutritional environments observed in laboratory studies with artificial diets, are useful to formulate predictions on the foraging and feeding behaviour and performance of insects in nature^6^. Based on our results, we propose that the ability of *A. ludens* to establish and thrive in novel host fruit could be restricted by the availability and ratio of nutrients in fruit, and by the presence of secondary metabolites in high concentrations in the context of particular nutrient ratios. *Anastrepha ludens* individuals exploiting host fruit with a high proportion of sugars relative to protein, could have longer larval and pupal developmental periods than their counterparts using host fruit with low proportion of sugar relative to protein, as predicted by our models (Fig. 1b,c). Similarly, longer developmental periods could be expected in host fruit with a high proportion of sugars relative to protein and a high content of certain phenolic compounds than in host fruit with a lower proportion of sugar relative to protein and a similar content of phenolics (Fig. 3). Interestingly, longer larval development times were reported when *A. ludens* developed in the apple cultivar ‘Fuji’ compared to larvae that developed in cultivar ‘Golden’^20^. ‘Fuji’ apples have a higher proportion of sucrose:protein than ‘Golden’ apples (‘Fuji’: 91.7:8.3; ‘Golden’: 88.1:11.9)^54^, and also a higher total content of phenolic compounds (‘Fuji’: 1.39 g/kg; ‘Golden: 1.21 g/kg)^20^.

Longer developmental periods are associated with a longer period of exposure to natural enemies and thus with a higher risk of mortality, as predicted by the slow-growth – high-mortality hypothesis^55^. *Anastrepha ludens* is attacked by several natural enemies including larval and pupal parasitoids^32^. Thus, we could expect a higher level of pupal parasitism in individuals from populations exploiting host fruit with a high proportion of sugars relative to protein and relatively high amounts of phenolic compounds, than in individuals exploiting host fruit with balanced or relatively low sugar content. Natural selection could favour individuals that escape parasitization or predation by developing faster as immatures in fruit with a low sugar:protein ratio. More research is required to test these types of predictions from studies using precisely defined diets, including the assessment of the abundance and ratios of nutrients in natural host fruit^6^.

The use of a mixture experiment approach in our study presented various advantages over more conservative ‘one variable at a time’ (OVAT) approach commonly used in animal nutritional biology studies. OVAT experiments consider explanatory variables measured on a continuous scale into factors, and the level of one factor is varied while the levels of other factors are kept constant^56^,^57^. This causes a loss of explanatory power in the analyses as it is only possible to make comparisons between the levels of the factors evaluated; predictions cannot be generated on levels not evaluated. For example, OVAT experiments were used to explore diet effects on the ability of pupating *C. capitata* larvae to accumulate protein and lipids, and on developmental indicators such as development time, pupation and adult production^19^. In a first experiment, yeast level in the diet varied while maintaining a constant level of sucrose; in the second experiment, sucrose level varied while keeping a constant level of yeast. This OVAT experiment allowed the identification of the yeast × sucrose combination(s) with the strongest effects on the response variables^19^. However, this design did not provide information on interactions and was unable to make predictions of dietary levels not evaluated. Unlike OVAT experiments, the mixture experiment we employed, allowed us to simultaneously examine yeast, sucrose, and their interactions, as well as to construct statistical models of use for evaluating the direction and contribution of each mixture component, and to make predictions concerning mixtures that had not been evaluated empirically.

Although we estimated the protein, lipid and carbohydrate input of yeast and sucrose mixtures to each of the diets tested, we were unable to separate the individual effects of nutrients within yeast (e.g., protein, lipids and carbohydrates). This is a caveat in our study that we clearly recognize, but that we believe does not invalidate the way we have interpreted our results. To address this issue, a fully chemically defined holidic diet is required to rear *A. ludens*. We also recognize that modifying the yeast:sucrose proportions in the diet affected diet consistency and texture, and this should be taken into consideration in future studies.

In summary, *A. ludens* was able to survive and develop to adulthood in 12 different diets across a wide range of nutrient ratios (Fig. 1, Table 1). Nevertheless, the nutritional balance of the larval diet induced changes in *A. ludens* phenotype across the larval, pupal and adult stages. With the exception of a potential trade-off between the duration of the pupal stage and adult resistance to desiccation and starvation, no physiological and developmental trade-offs were detected. The nutrient balance of the larval diet conditioned the effects of chlorogenic acid on *A. ludens* larval development. Further studies should look into this topic and explore the interactions between different food components, modulating *A. ludens* phenotype across life stages and mediating interactions between *A. ludens* and other organisms. This is at the centre of nutritional ecology^24^,^58^, and is a major challenge in nutritional immunology^59^. The experimental and modelling approach reported here could be helpful in achieving these goals and contribute to generating predictive models of life history traits in a wide variety of organisms. We conclude that nutritional and non-nutritional food components in the larval diet of this devastating fruit fly pest have cascading effects across the immature and adult stages and could mediate interactions affecting the fly’s ability to establish and thrive in novel hosts.

## Additional Information

**How to cite this article**: Pascacio-Villafán, C. *et al.* Nutritional and non-nutritional food components modulate phenotypic variation but not physiological trade-offs in an insect. *Sci. Rep.* **6**, 29413; doi: 10.1038/srep29413 (2016).

## Supplementary Material

Supplementary Information

## Figures and Tables

**Table 1:** Nutritional and calorie contribution of yeast:sucrose mixtures to experimental diets in Experiment 1 (D1-D17), and Experiment 2 (low yeast-high sucrose [LY-HS] and high yeast-low sucrose [HY-LS]).

Mixture No.	Yeast (%)	Sucrose (%)	Protein (%)^a^	(Lipid (%))^a^	Carbohydrate (%)^a^	Calories (kcal/3.55 g)^a^
D1	0	100	0.014	0	14.18	14.20
D2	3	97	0.206	0.0004	13.91	14.12
D3	7	93	0.461	0.0009	13.55	14.01
D4	12	88	0.781	0.0017	13.09	13.87
D5	24	76	1.548	0.0034	11.99	13.55
D6	32	68	2.059	0.0045	11.27	13.33
D7	38	62	2.442	0.0053	10.72	13.17
D8	42	58	2.711	0.0059	10.34	12.92
D9	50	50	3.209	0.0071	9.63	12.85
D10	58	42	3.708	0.0082	8.92	12.52
D11	62	38	3.976	0.0088	8.53	12.53
D12	68	32	4.359	0.0096	7.99	12.37
D13	76	24	4.871	0.0107	7.26	12.15
D14	88	12	5.637	0.0124	6.16	11.83
D15	93	7	5.957	0.0132	5.71	11.69
D16	97	3	6.212	0.0137	5.34	11.59
D17	100	0	6.404	0.0142	5.07	11.51
LY-HS	34	66	2.219	0.0049	11.04	13.28
HY-LS	55	45	3.524	0.0078	9.18	12.72

^a^Estimated from separate bromatological analyses of yeast and sucrose (Supplementary Table S1).

**Table 2:** ANOVA and summary statistics for models fitted to data on larval survival to pupa, duration of the larval stage, pupal weight, adult emergence, duration of the pupal stage and adult resistance to desiccation and starvation of *Anastrepha ludens* reared on artificial diets with varying proportions of yeast (Y) and sucrose (S).

Response variables^a^	ANOVA				R^2^	R^2^_adj_	R^2^_pred_
	Model^b^	Linear mixture^c^	Y × S	Lack of fit^d^			
Survival to pupa	F = 53.09 P < 0.0001	F = 0.35 P = 0.5568	F = 105.83 P < 0.0001	F = 1.45 P = 0.1555	0.56	0.55	0.54
Duration of the larval stage	F = 56.96 P < 0.0001	F = 92.34 P < 0.0001	F = 27.04 P < 0.0001	F = 0.61 P = 0.7799	0.75	0.74	0.72
Pupal weight	F = 5.24 P = 0.0096	F = 4.39 P = 0.0426	F = 6.09 P = 0.018	F = 0.091 P = 0.9996	0.21	0.17	0.12
Adult emergence	F = 3.76 P = 0.0595	F = 3.76 P = 0.0595	—	F = 0.32 P = 0.9683	0.08	0.06	0.002
Duration of the pupal stage	F = 133.33 P < 0.0001	F = 133.33 P < 0.0001	—	F = 1.78 P = 0.1091	0.77	0.76	0.73
Desiccation and starvation resistance	F = 64.56 P < 0.0001	F = 61.05 P < 0.0001	F = 68.06 P < 0.0001	F = 1.02 P = 0.4478	0.76	0.75	0.72

^a^Prior to model fitting, data on adult emergence were normalized by a logit transformation; data from all other response variables were modelled without transformation.

^b^Linear models were fitted to data on adult emergence and on the duration of the pupal stage; quadratic models were fitted to all other response variables.

^c^The linear mixture compares the response at the extreme ends of the model.

^d^Variation of the data around the fitted model.

**Table 3:** ANOVA and summary statistics for models fitted to data on the duration of the larval stage, duration of the pupal stage, pupal weight, survival to adult, development time to adulthood and adult resistance to desiccation and starvation of *Anastrepha ludens* reared on low yeast-high sucrose and high yeast-low sucrose content diets (Diet) treated with chlorogenic acid (C. acid).

Response variables^a^	ANOVA					R^2^	R^2^_adj_	R^2^_pred_
	Model^b^	Diet	C. acid	Diet × C. acid	Lack of fit^c^			
Duration of the larval stage	F = 7.46 P = 0.0019	F = 15.22 P = 0.001	F = 4.05 P = 0.0594	F = 4.67 P = 0.0443	F = 0.84 P = 0.3741	0.55	0.48	0.31
Duration of the pupal stage	—	—	—	—	F = 1.99 P = 0.1347	0.0	0.0	−0.09
Pupal weight	—	—	—	—	F = 1.32 P = 0.3044	0.0	0.0	−0.09
Survival to adult	—	—	—	—	F = 0.45 P = 0.8088	0.0	0.0	−0.09
Development time to adulthood	F = 7.84 P = 0.0111	F = 7.84 P = 0.0111	—	—	F = 1.44 P = 0.2664	0.28	0.25	0.13
Desiccation and starvation resistance	—	—	—	—	F = 1.36 P = 0.2892	0.0	0.0	−0.09

^a^Prior to model fitting, data on pupal weight and development time to adulthood were normalized by rank transformation. Data from all other response variables were modelled without transformation.

^b^A mean model was fitted to data on the duration of the pupal stage (after reduction from a 2-factor interaction model), pupal weight (after reduction from a linear model), survival to adult and adult resistance to desiccation and starvation (after reduction from a quadratic model); a reduced linear model was fitted to data on development time to adulthood; a 2-factor interaction model was fitted to data on the duration of the larval stage.

^c^Variation of the data around the fitted model.
