# Supplementary material for: Nutritional and non-nutritional food components modulate phenotypic variation but not physiological trade-offs in an insect
Source: Sci Rep. 2016 Jul 12;6:29413. doi: 10.1038/srep29413 (PMC4996112; doi:10.1038/srep29413)
Supplement: Supplementary Information [file srep29413-s1.pdf]

Supplementary Information

Nutritional and non-nutritional food components modulate phenotypic variation but not physiological trade-offs in an insect

Carlos Pascacio-Villafán[1,*], Trevor Williams[1], Andrea Birke[1], and Martín Aluja[1,†]

[1] Instituto de Ecología, A.C., Red de Manejo Biorracional de Plagas y Vectores, Xalapa 91070, Veracruz, Mexico

[*] cpascacio@hotmail.com
[†] martin.aluja@inecol.mx

Supplementary Figures S1 and S2. Exploratory Boxplots.

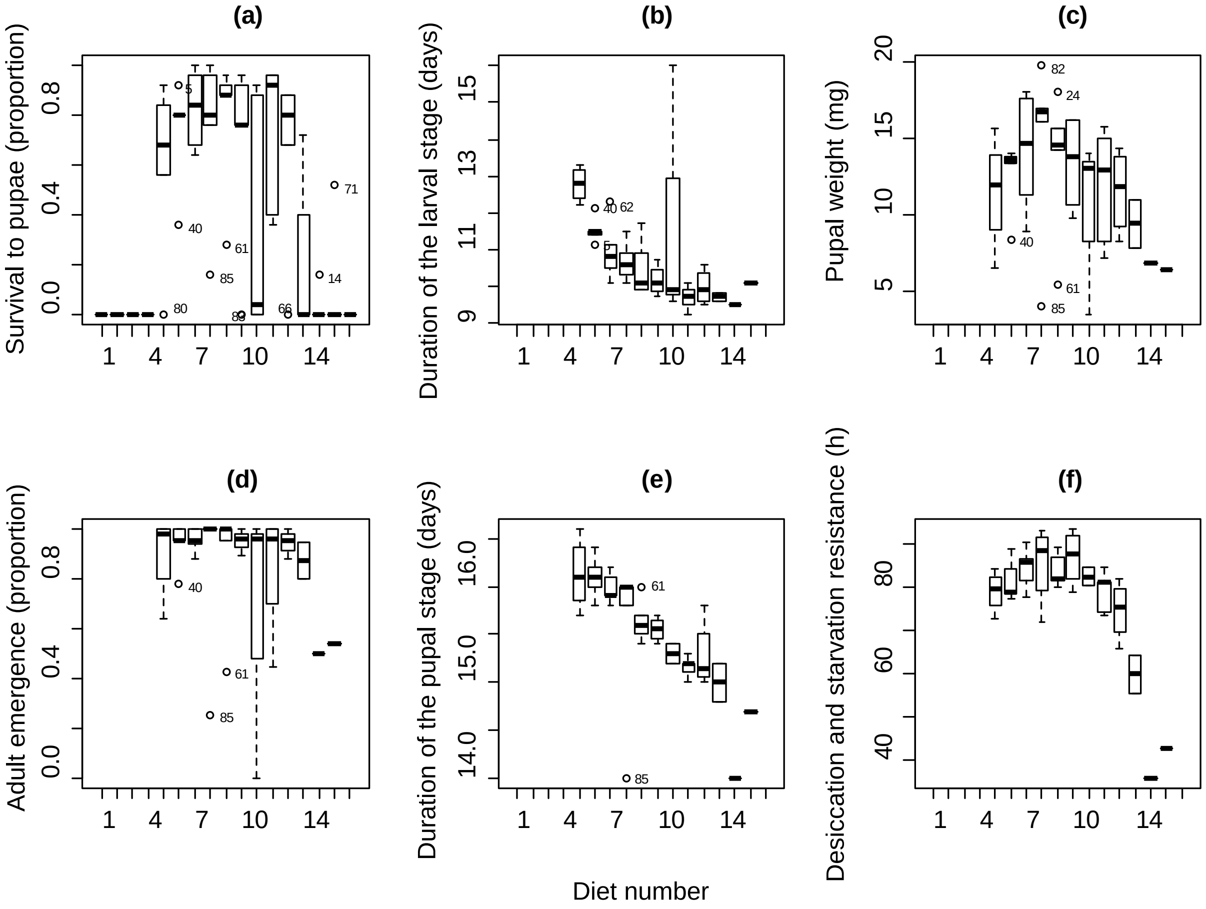

Figure S1 Boxplots of: (a) survival to pupa (proportion), (b) duration of the larval stage (days), (c) pupal weight (mg), (d) adult emergence (proportion), (e) duration of the pupal stage (days), and (f) desiccation and starvation resistance (hours) of *Anastrepha ludens* reared in 17 diets (D1 - D17) with varying yeast:sucrose proportions (Table 1 in main text). Boxes extend from the 25% to the 75% quartile, and the horizontal line in each box indicates the median. Whiskers indicate the minimum and maximum values; if there are outliers (circles beyond whiskers), the whiskers indicate 1.5 times the size of the hinge, which is the 75% minus 25% quartiles. Numbers next to circles indicate the corresponding run number (Supplementary Table S1).

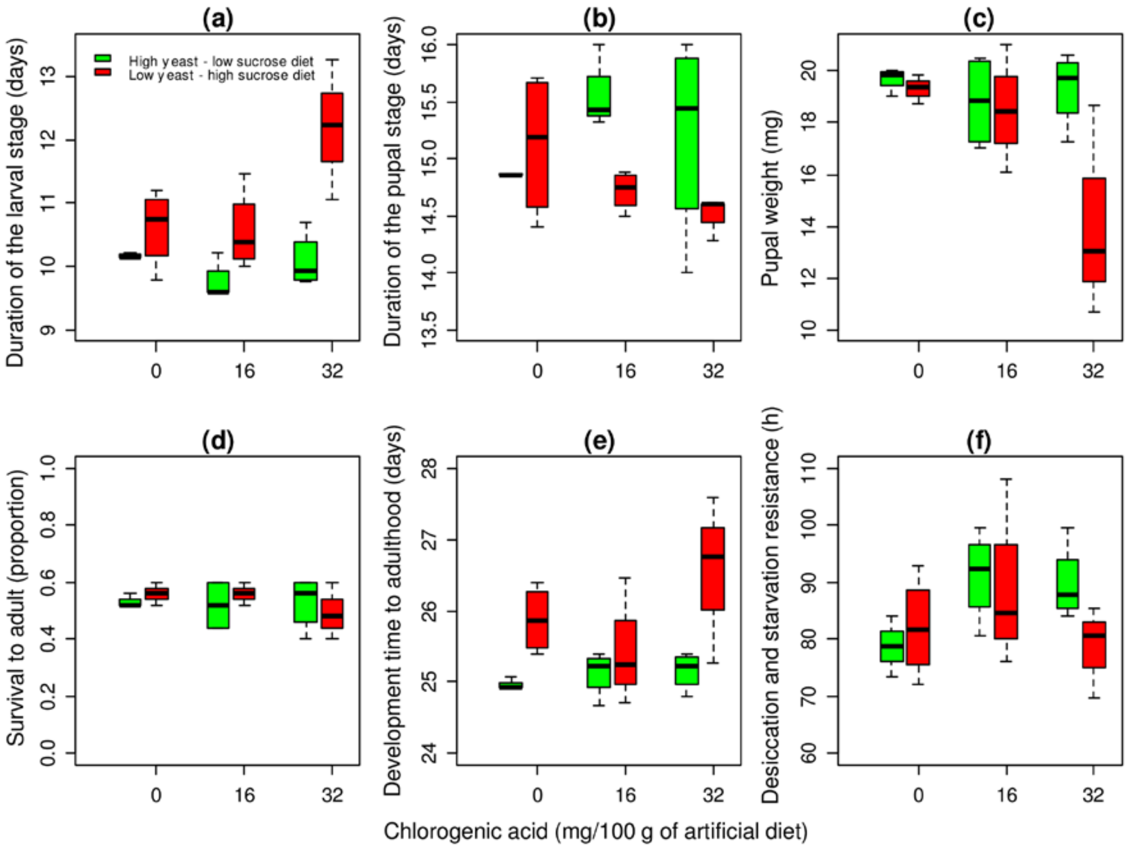

Figure S2 Boxplots of: (a) duration of the larval stage (days), (b) duration of the pupal stage (days), (c) pupal weight (mg), (d) survival to adult (proportion), (e) development time to adulthood (days), and (f) desiccation and starvation resistance (hours) of *Anastrepha ludens* reared in high yeast – low sucrose (green boxes) and low yeast – high sucrose (red boxes) diets treated with chlorogenic acid at 0, 16 and 32 mg/100 g of artificial diet. Each box extends from the 25% to the 75% quartile, and the line in the box indicates the median. Whiskers indicate the range.

Supplementary Figures S3-S12. Diagnostic and influence plots, and model discussion.

EXPERIMENT 1

Survival to pupa

We fitted a quadratic model to untransformed data and considered this as the model that provided the most suitable description of the data on survival to pupa (Fig. 1a in main text). Diagnostic plots are shown in Fig. S3. The normal plot of residuals showed no mayor deviation of the points around the line. The variance scatter in the residuals vs. predicted plot revealed a linear trend that represents the mixtures with zero pupal survival. This pattern is the result of lack of variability in mixtures with no pupal survival. The model was inadequate in predicting survival to pupae, especially when survival was close to zero, as observed in the plot of predicted vs. actual values. A Box-Cox plot for power transformations, which shows the current (blue line), best (green line) and 95% confidence range (red lines) lambda values, suggested a log (y + 0.001) transformation but this was not applied because of unrealistic model estimates of proportions of survival that exceeded 1. No overly influential data points were identified in DFFITS and DFBETAS plots. The externally studentized residuals and Cook's distance plots did not reveal outlier points.

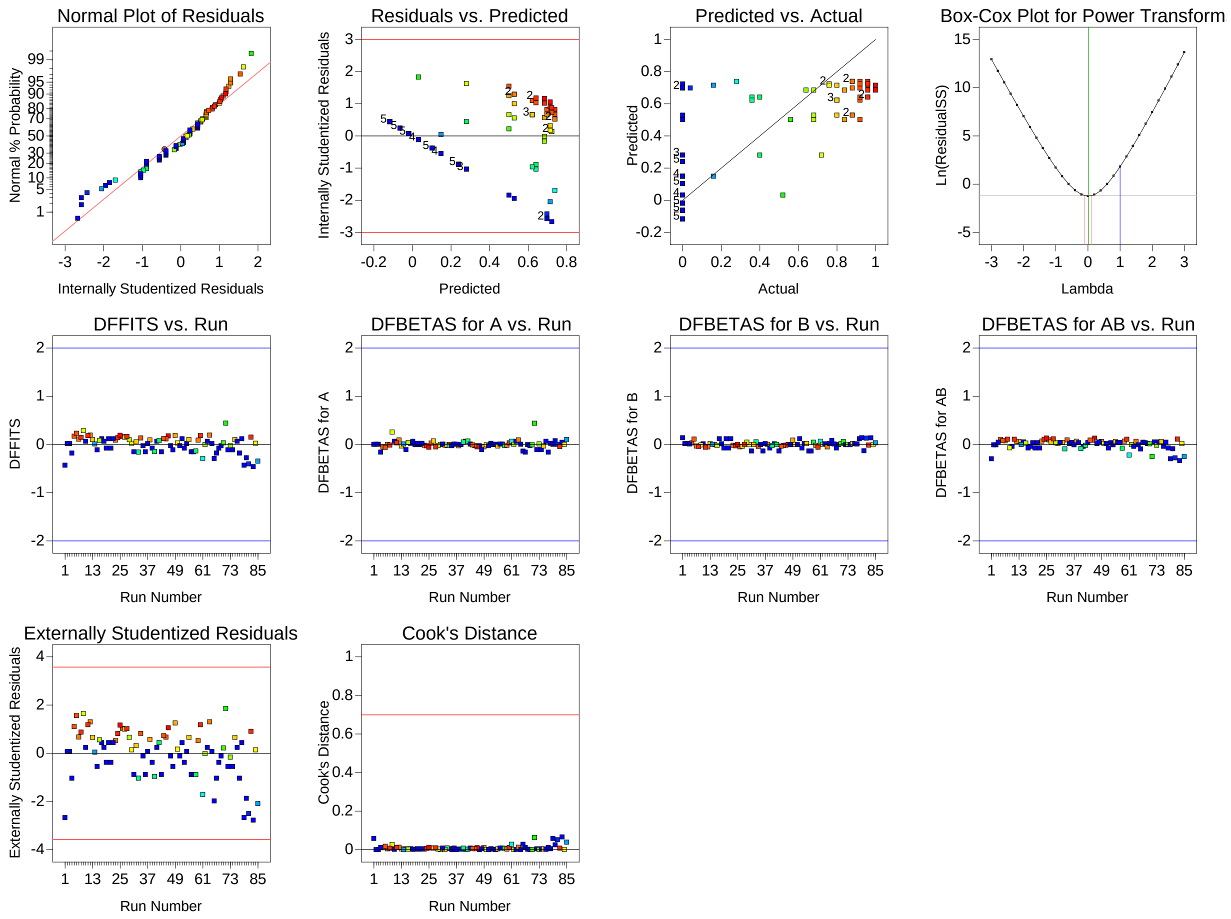

Figure S3 Diagnostic plots of a quadratic model fitted to data on survival to pupae (proportion) of *Anastrepha ludens* flies reared on diets with varying proportions of yeast (A) and sucrose (B). The colour of the points indicate the value of proportion of larvae that pupated, ranging from zero (blue) to 1 (red).

Duration of the larval stage

A linear model was first fitted to the data on the duration of the larval stage (days) (Model: $F = 18.25$, $P < 0.001$; Lack of fit: $F = 1.37$, $P = 0.2398$; yeast $\beta = 9.05$, 95% CI: 8.18, 9.92; sucrose $\beta = 12.66$, 95% CI: 11.70, 13.63; $R^2 = 0.3029$, $R^2_{adj} = 0.2863$, $R^2_{pred} = 0.2506$). Diagnostic and influence plots are shown in Fig. S4. Run 81 was identified as an outlier and a highly influential data point by the normal plot of residuals, the residuals vs. predicted values plot and the externally studentized residuals plot. A Box-Cox plot did not indicate the need for transformation. Run 81 was further investigated to determine whether it could be attributed to a particular biological cause. Run 81 corresponds to a Petri dish in which only one larva pupated, out of a group of 25 insects. The time it took for that larva to pupate was highly inconsistent with larval duration observed in the remaining runs of the same dietary treatment. Furthermore, the larva in question did not transform into a viable pupa (i.e., it was a pupa from which no adult emerged). This atypical observation raised the suspicion that there may have been an issue with the experimental validity of run 81. Therefore, the following analyses were performed having excluded run 81 from the dataset.

The model of best fit to describe the duration of the larval stage (ignoring run 81) was quadratic (Fig. 1b in main text). Diagnostic and influence plots are shown in Fig. S5. Ignoring run 81 greatly improved diagnostic and influence plots (Fig. S5). The normal plot of residuals indicated no deviation from normality. The residual vs. predicted plot indicated no serious distortion that would suggest heteroscedasticity. The predicted vs. actual plot indicated that the predictive accuracy of the model was highest between 9 and 12 days. A Box-Cox plot suggested a power transformation (lambda = -2.36). However, fitting a quadratic relationship to transformed data (duration of the larval stage$^{-2.36}$) did not result in a model with improved explanatory power (Model: $F = 55.96$, $P < 0.0001$; Linear mixture: $F = 92.29$, $P < 0.0001$; yeast x sucrose: $F = 19.63$, $P < 0.0001$), or marked improvements in any of the diagnostic plots. Therefore, we retained the model based on untransformed data. No overly influential data points were detected in DFFITS and DFBETAS plots. The externally studentized residuals and Cook´s distance plots did not reveal outlier points. The quadratic model fitted to untransformed data (excluding run 81) did not change the direction of the yeast and sucrose effects observed in the linear model that included run 81. It did, however, improve the explanatory power of the model and allow model assumptions of normality and equal variance to be met (Fig. S5). The conclusions of the analyses from both models were similar. Therefore, we conclude that the quadratic model ignoring run 81 provided a better description of the trends in the data than did the linear model that included run 81. For the above mentioned reasons, run 81 was also excluded from all subsequent analyses of response variables.

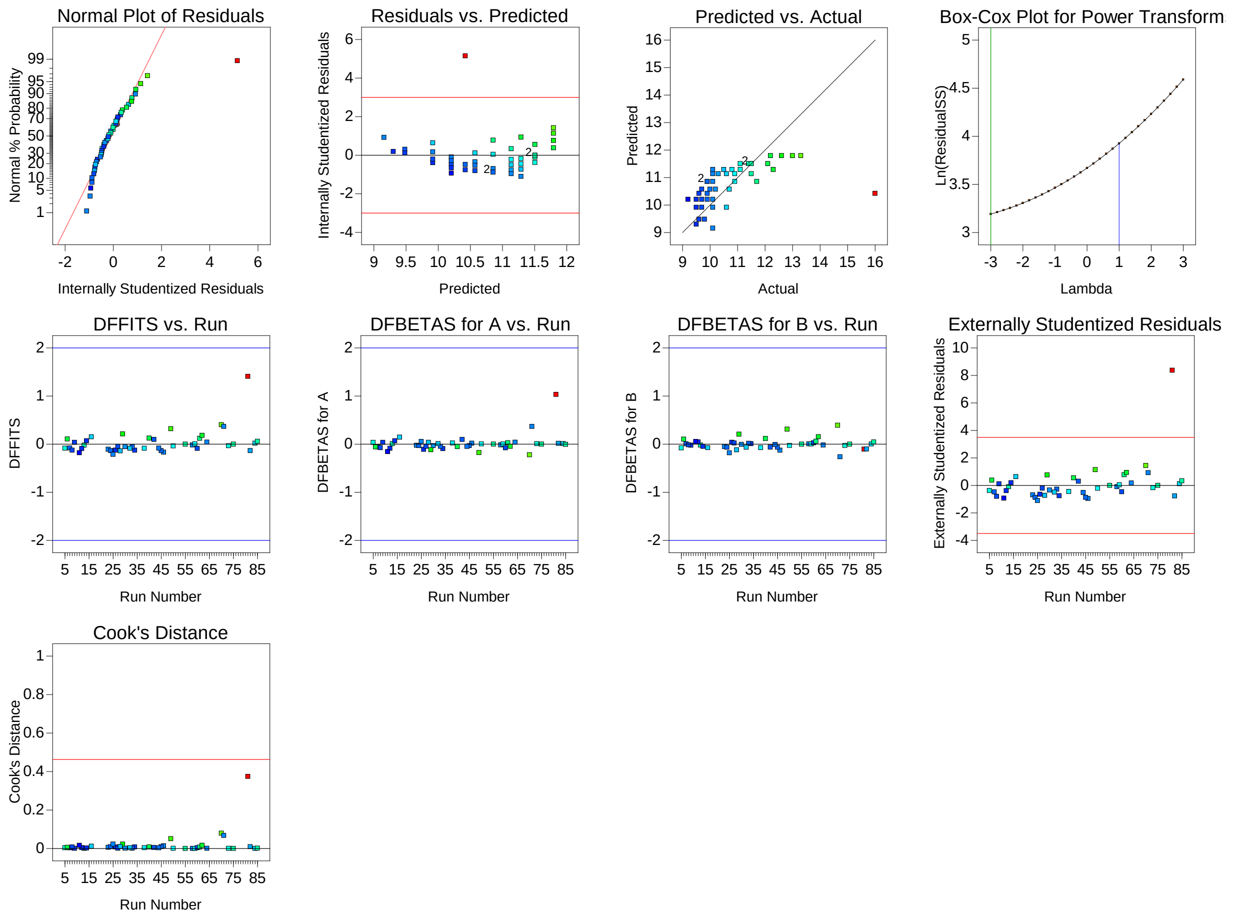

Figure S4 Diagnostic plots of a linear model fitted to data on duration of the larval stage (days) of *Anastrepha ludens* flies reared on diets with varying proportions of yeast and sucrose. The colour of the points indicate the mean duration of the larval stage, ranging from 9.2 days (blue) to 16 days (red).

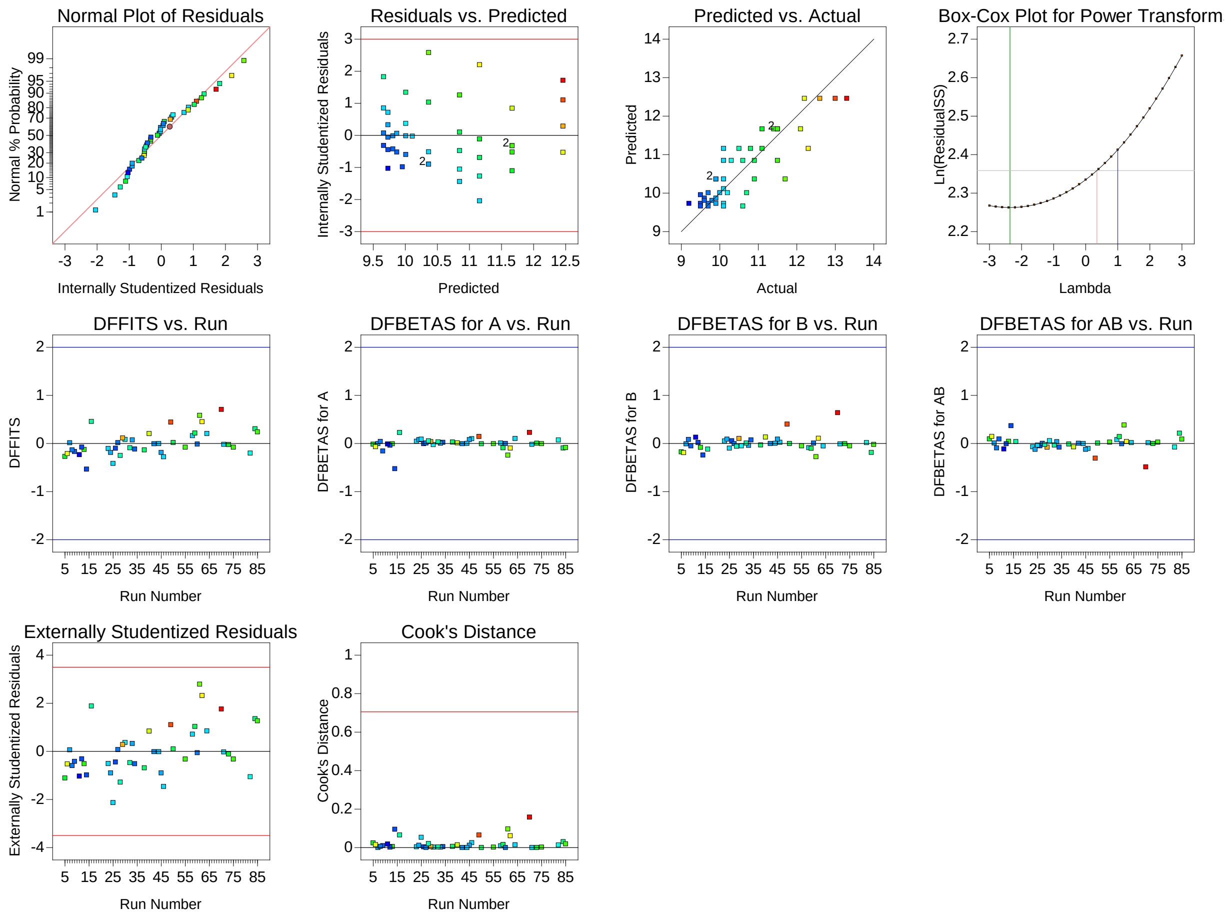

Figure S5 Diagnostic plots of a quadratic model fitted to data on duration of the larval stage (days) of *Anastrepha ludens* flies reared on diets with varying proportions of yeast and sucrose. The colour of the points indicate the mean duration of the larval stage, ranging from 9.2 days (blue) to 13.3 days (red).

Pupal weight

The model of best fit to pupal weight data (excluding run 81) was quadratic (Fig. 1c in main text). Diagnostic plots are shown in Fig. S6. The normal plot of residuals showed no marked deviation from normality. The pattern observed in the residuals vs. predicted plot was caused by a lack of values towards mixtures approaching pure yeast (Fig. 1c in main text), i.e., there were only between one and two data points at these mixtures, whereas other mixtures had four or five data points. As such, there was a low number of low predicted values. The predicted vs. actual plot indicated that the model was not capable of forming predictions on pupal weight with a useful degree of confidence. A Box-Cox plot did not indicate the need for transformation. There were no other noteworthy issues with the influence plots (DFFITS, DFBETAS, externally studentized residuals and Cook's distance plots).

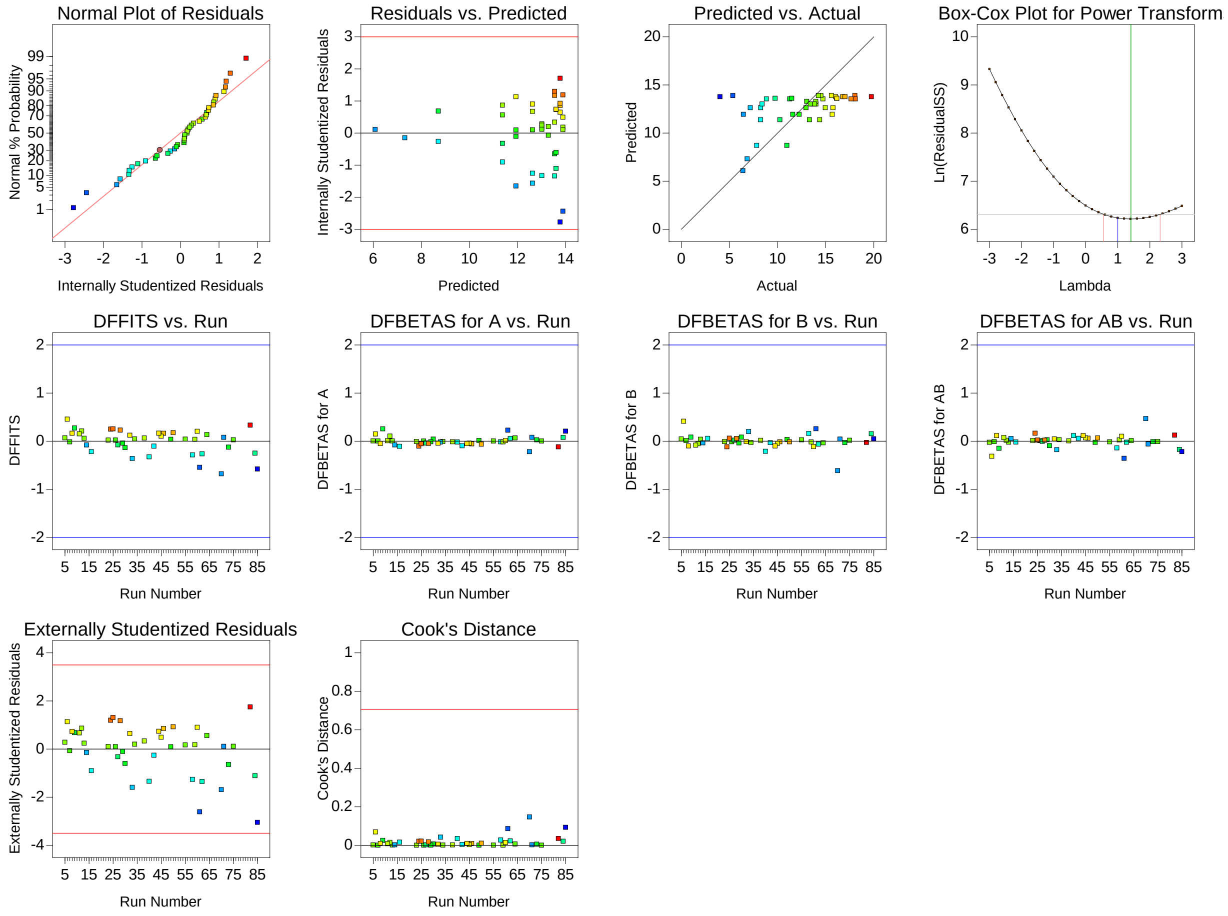

Figure S6 Diagnostic plots of a quadratic model fitted to data on mean pupal weight (mg) of *Anastrepha ludens* flies reared on diets with varying proportions of yeast and sucrose. The colour of the points indicate the mean pupal weight, ranging from 4.03 mg (blue) to 19.76 days (red).

Adult emergence

The model of best fit to data on logit transformed proportions of adult emergence (excluding run 81) was linear (Fig. 1d in main text). Diagnostic plots are shown in Fig. S7. The normal plot of residuals showed that overall the points clustered close to the line. No serious distortion that would suggest heteroscedasticity was observed in the plot of residuals vs. predicted values. The predicted values vs. actual values plot indicated that the model is not useful for prediction. Box-Cox plot is not applicable for logit transformed values. No overly influential data points were identified in DFFITS and DFBETAS plots. The externally studentized residuals and Cook's distance plots did not reveal outlier points.

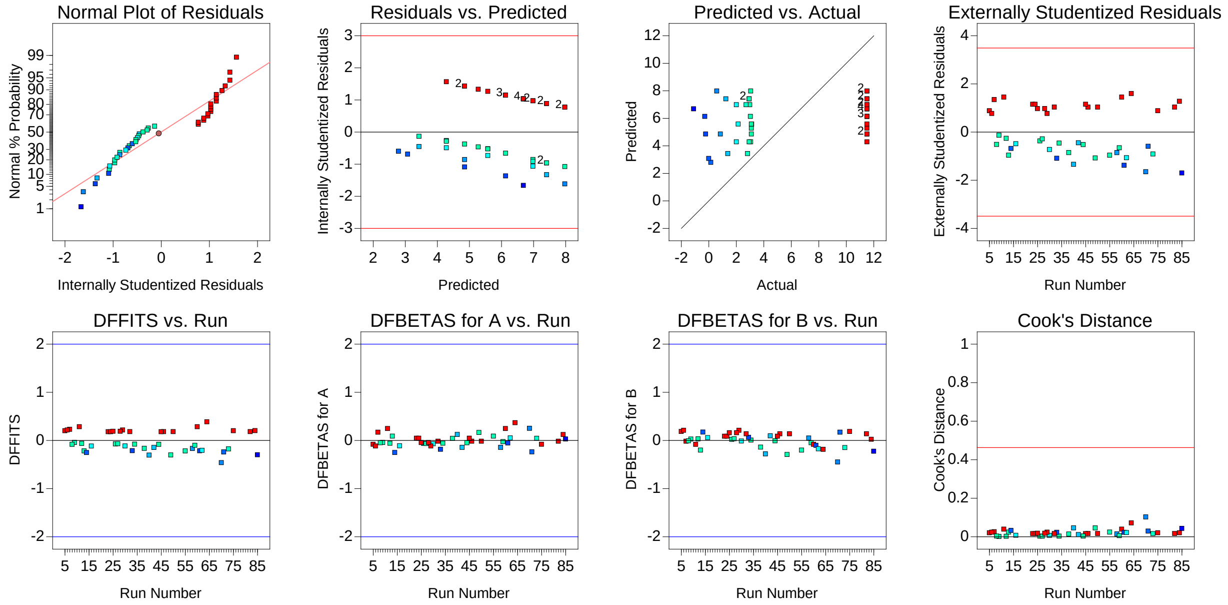

Figure S7 Diagnostic plots of a linear model fitted to logit transformed values on adult emergence (proportion) of *Anastrepha ludens* flies reared on diets with varying proportions of yeast and sucrose. The colour of the points indicate the proportion of adult emergence, ranging from 0.5 (blue) to 1 (red).

Duration of the pupal stage

A linear model was fitted to the data on the duration of the pupal stage (days) (Model: $F = 53.16$, $P < 0.001$; Lack of fit: $F = 0.80$, $P = 0.6295$; yeast $\beta = 14.57$, 95% CI: 14.27, 14.87; sucrose $\beta = 16.68$, 95% CI: 16.35, 17.01; $R^2 = 0.5646$, $R^2_{adj} = 0.5540$, $R^2_{pred} = 0.5203$). Diagnostic and influence plots are shown in Fig. S8. The normal plot of residuals showed a deviation of the points from a normal distribution. One point identified as run 85 with an internally studentized residual value of -4.8, was particularly far from the line. The residuals vs. predicted plot showed that the same point, run 85, fell outside the interval of −3 to +3 standard deviations. The predicted vs. actual plot indicated a good predictive accuracy of the model, however, run 85 again lay far beyond the predicted line. A Box-Cox plot did not suggest the need for transformation. No overly influential data points were identified in DFFITS and DFBETAS plots. The externally studentized residuals plot revealed run 85 as an outlier point. We modelled the data again to assess whether ignoring this atypical and seemingly highly influential observation could lead to meet the model assumptions of normality and homoscedasticity.

A linear model fitted to data on the duration of the pupal stage that excluded run 85 (Fig. 1e in main text), provided a substantial improvement in the diagnostic and influence plots (Fig. S9). The conclusions of this model were not different from the conclusions of the linear model that included run 85. The normal plot of residuals showed no marked deviation from normality. There was no serious distortion in the residuals vs. predicted values plot that suggested lack of constant variance; however, there were two points (runs 64 in the top and 14 in the bottom) that fell near or on the three sigma limits (red horizontal lines). The predicted values vs. actual values plot indicated that the model was useful for prediction as points were distributed evenly above and below the fitted line. A Box-Cox plot did not indicate the need for transformation. No overly influential data points were identified in DFFITS and DFBETAS plots. The externally studentized residuals plot did not reveal influential outliers, whereas the Cook's distance plot identified run 14 as an outlier. We were unable to identify the reasons behind runs 14 and 64 being identified as outlier observations. A linear model fitted to data ignoring runs 14 and 64 did not provide a different interpretation of the results ($F = 171.99$, $P < 0.0001$; yeast $\beta = 14.60$; 95% CI: 14.42, 14.77; sucrose $\beta = 16.74$; 95% CI: 16.56, 16.93). Therefore, we retained the linear model with runs 14 and 64 (but excluding run 85) as the best model to describe the data.

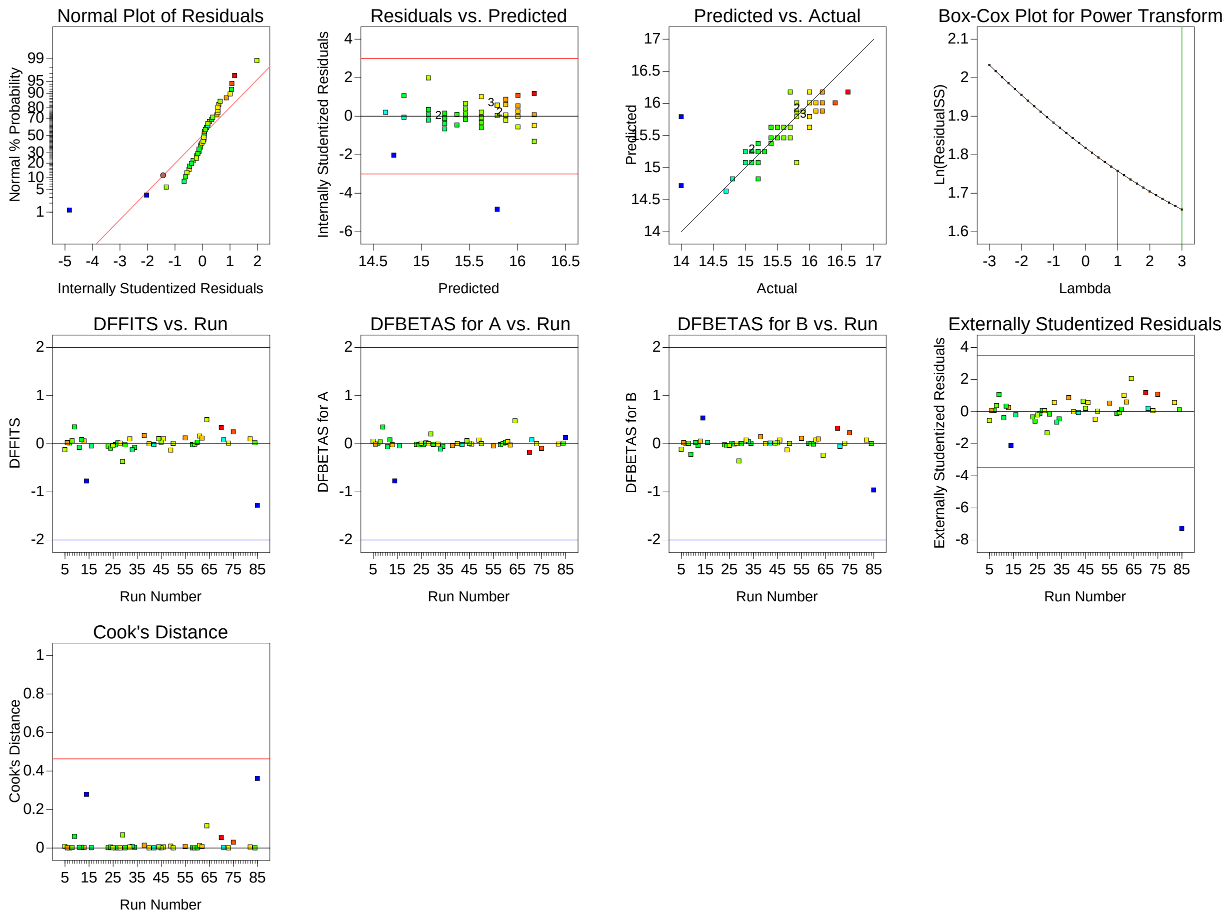

Figure S8 Diagnostic plots of a linear model fitted to data on the duration of the pupal stage (days) of *Anastrepha ludens* flies reared on diets with varying proportions of yeast and sucrose. The colour of the points indicate the duration of the pupal stage, ranging from 14 days (blue) to 16.6 days (red).

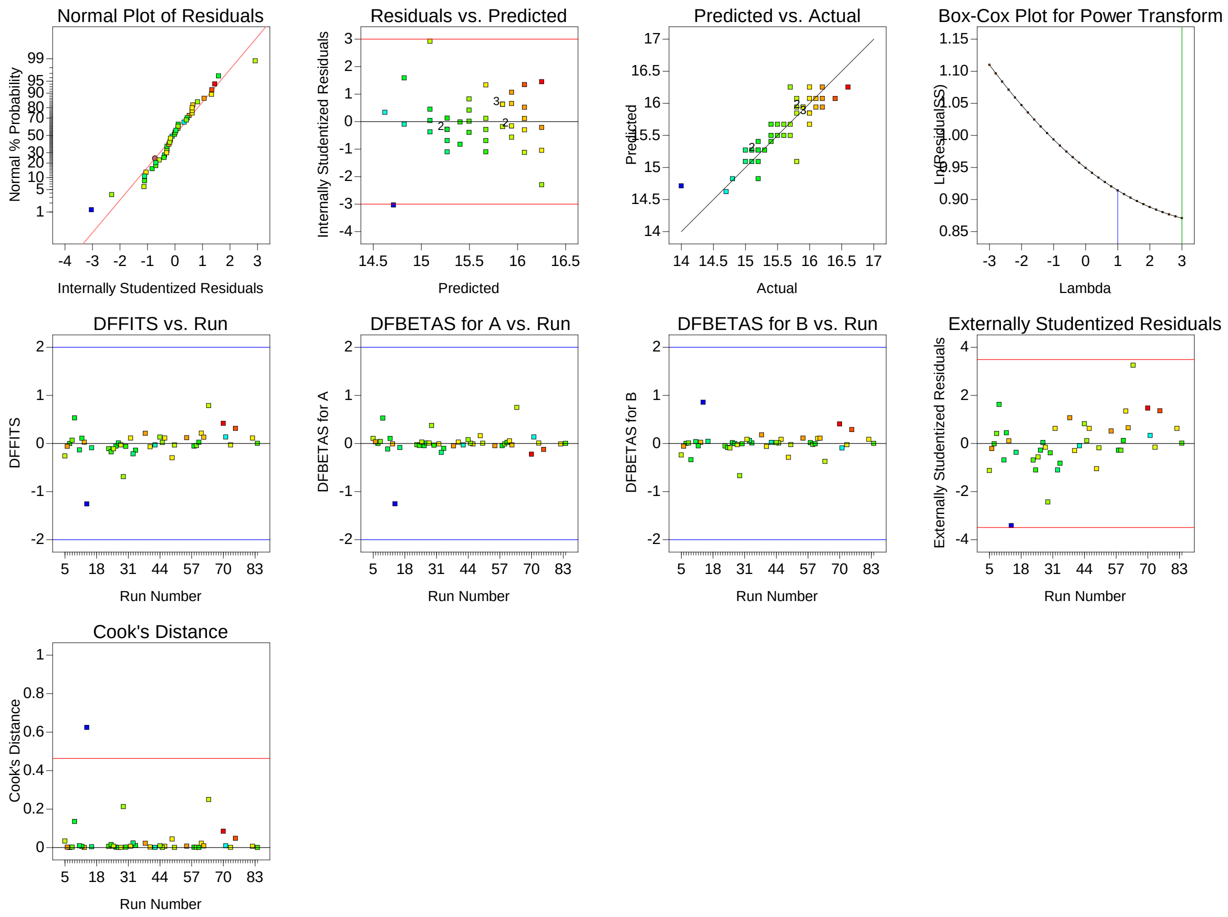

Figure S9 Diagnostic plots of a linear model fitted to data on the duration of the pupal stage (days) of *Anastrepha ludens* flies reared on diets with varying proportions of yeast and sucrose. The colour of the points indicate the duration of the pupal stage, ranging from 14 days (blue) to 16.6 days (red).

Desiccation and starvation resistance

The model of best fit for desiccation and starvation resistance data was quadratic (Fig. 1f in main text). Diagnostic plots are shown in Fig. S10. No marked deviation from normality was detected; there are small curvatures along the line but no obvious distortion. The variance appeared constant. The predicted vs. actual values plot indicated that the model was useful for prediction as the points are equally distributed above and below the fitted line. A Box-Cox plot did not indicate the need for transformation. No overly influential data points were identified in DFFITS and DFBETAS plots. The externally studentized residuals and Cook's distance plots did not reveal outlier points.

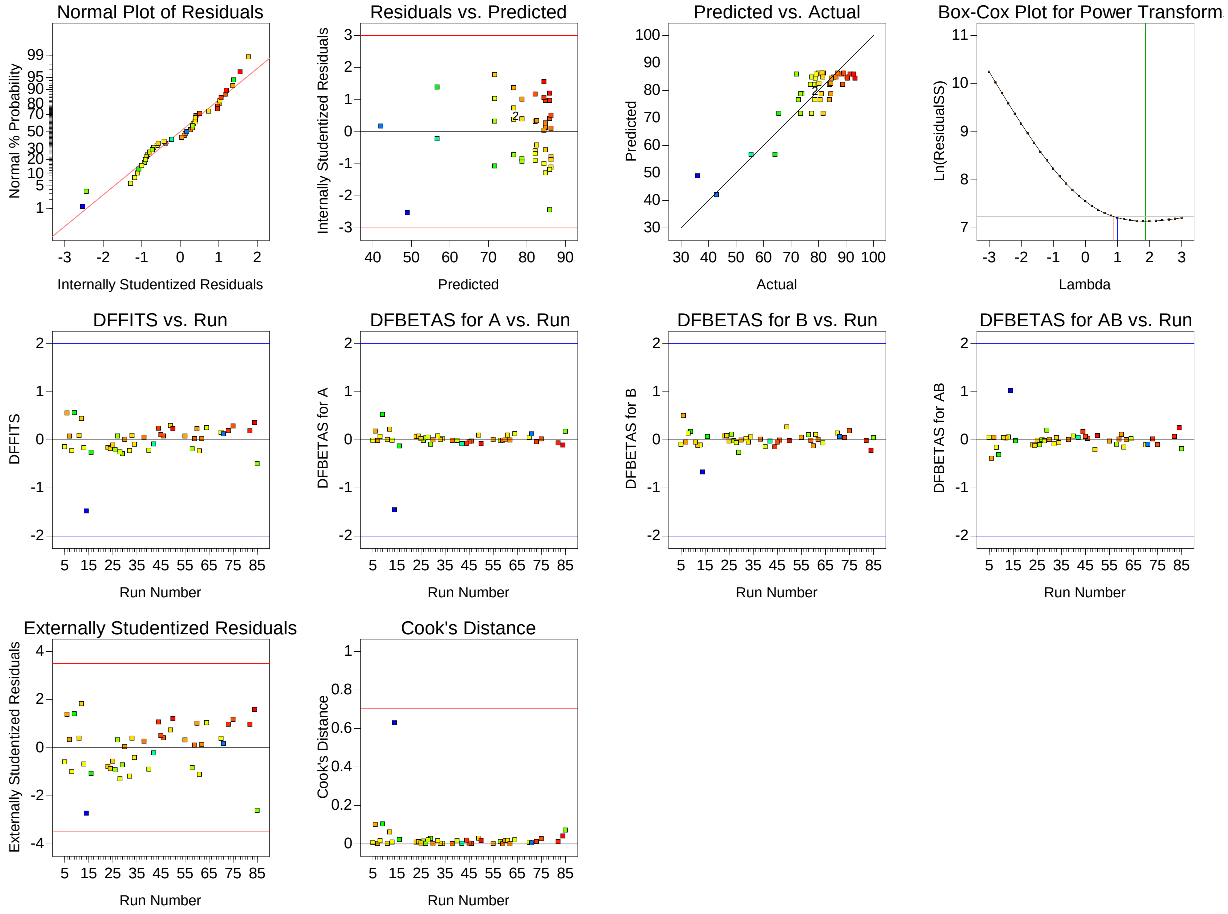

Figure S10 Diagnostic plots of a quadratic model fitted to data on adult resistance to starvation (h) of *Anastrepha ludens* flies reared on diets with varying proportions of yeast and sucrose. The colour of the points indicate the time adults survived without food, ranging from 36 h (blue) to 93.3 h (red).

EXPERIMENT 2

Duration of the larval stage

The best fit model to data on the duration of the larval stage was a two-factor interaction model (Fig. 3 in main text). Diagnostic plots are shown in Fig. S11. The normal plot of residuals showed no marked deviation from normality. The variance appeared constant in the residual vs. predicted values plot. The predicted vs. actual plot indicated that the model was better for prediction between 9 and 12 days. A Box-Cox plot did not indicate the need for transformation. No overly influential data points were identified in DFFITS and DFBETAS plots. The externally studentized residuals and Cook's distance plots did not reveal outlier points.

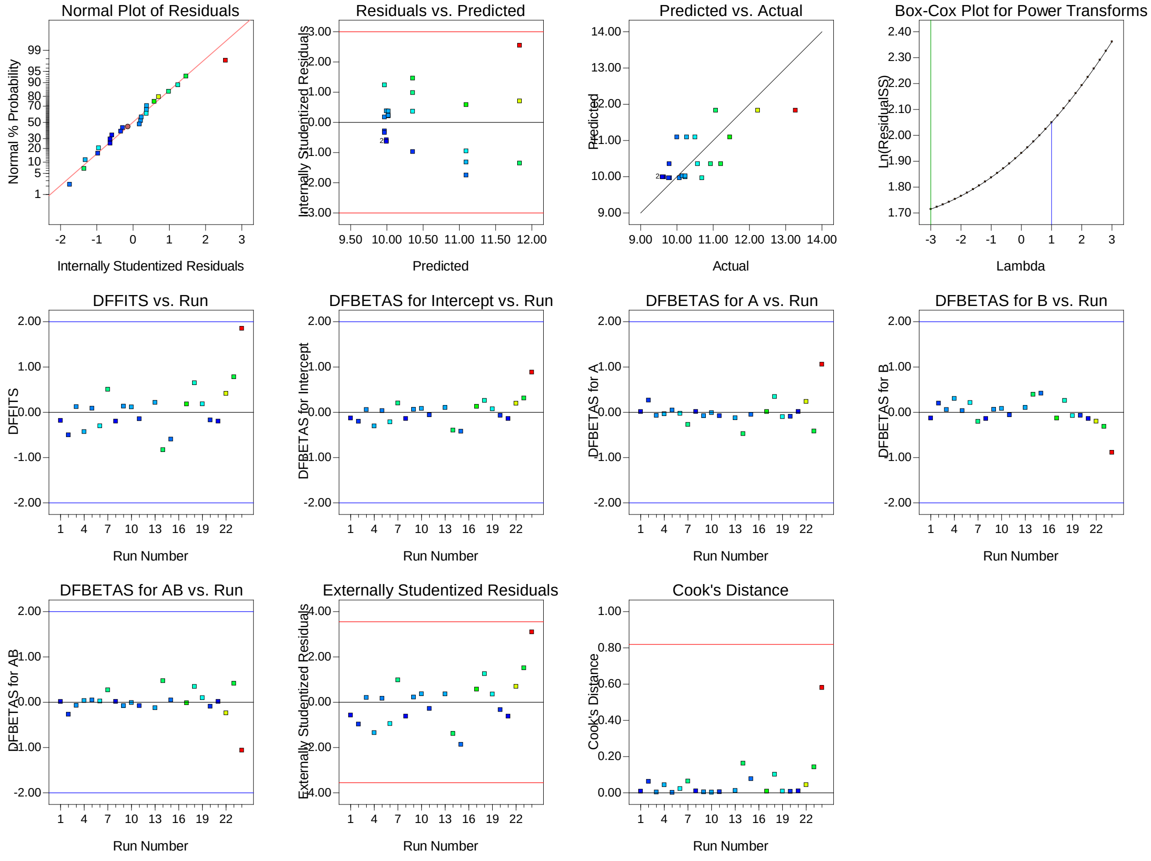

Figure S11 Diagnostic plots of a two-factor interaction model fitted to data on the duration of the larval stage (days) of *Anastrepha ludens* flies reared on diets with varying yeast:sucrose balance and chlorogenic acid content. The colour of the points indicate the duration of the larval stage, ranging from 9.6 days (blue) to 13.27 days (red).

Development time to adulthood

The best fit model for rank transformed data on the development time to adulthood was linear. Diagnostic plots are shown in Fig. S12. The normal plot of residuals showed no marked deviation from normality. The variance appeared constant. The predicted vs. actual values plot indicated a poor predictive capacity of the model. A Box-Cox plot did not indicate the need for transformation. No overly influential data points were identified in DFFITS and DFBETAS plots. The externally studentized residuals and Cook's distance plots did not reveal outlier points.

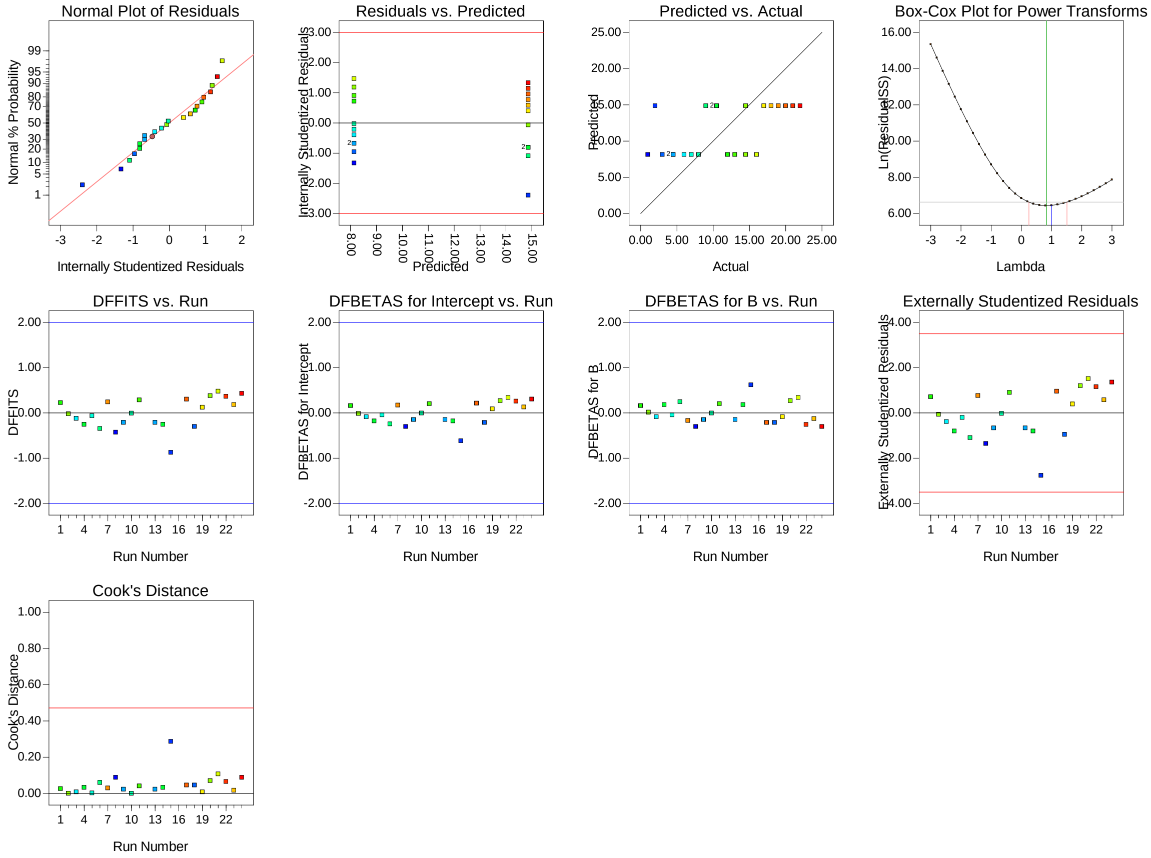

Figure S12 Diagnostic plots of a two-factor interaction model fitted to rank transformed data on the development time to adulthood (days) of *Anastrepha ludens* flies reared on diets with varying yeast:sucrose balance and chlorogenic acid content. The colour of the points indicate the development time to adulthood, ranging from 24.7 days (blue) to 27.6 days (red).

Supplementary Tables S1-S3.

Table S1 Estimation of nutrient and calorie contribution of yeast:sucrose mixtures to experimental diets tested in Experiment 1(D1-D17) and Experiment 2 (Low yeast-high sucrose [LY-HS] and High yeast-low sucrose [HY-LS]), based on bromatological analyses of yeast (protein: 45.1%, lipid: 0.1%, carbohydrate: 35.7%, calorie: 324.1 kcal/100 g) and sucrose (protein: 0.1%, lipid:ND, carbohydrate: 99.888%, calorie: 399.9 kcal/100 g).

Diet Number[a]	Yeast:Sucrose mixtures						Nutrient (%) and calorie (kcal) from individual yeast and sucrose[e]							Nutrient content (%) and calorie contribution of yeast:sucrose mixtures to the diets[f]			
	Mixture experiment[b]		Weight present in each diet[c]		% by total weight of diet[d]		Yeast				Sucrose						
	Yeast (%)	Sucrose (%)	Yeast (g)	Sucrose (g)	Yeast (%)	Sucrose (%)	Prot	Lip	Carb	kcal	Prot	Carb	kcal	Prot	Lip	Carb	kcal/3.55 g
D1	0	100	0.00	3.55	0.00	14.20	0.0000	0.0000	0.0000	0.0000	0.0142	14.1841	14.1965	0.0142	0.0000	14.1841	14.1965
D2	3	97	0.11	3.44	0.43	13.77	0.1921	0.0004	0.1521	0.3452	0.0138	13.7586	13.7706	0.2059	0.0004	13.9107	14.1157
D3	7	93	0.25	3.30	0.99	13.21	0.4483	0.0010	0.3549	0.8054	0.0132	13.1912	13.2027	0.4615	0.0010	13.5461	14.0081
D4	12	88	0.43	3.12	1.70	12.50	0.7685	0.0017	0.6083	1.3807	0.0125	12.4820	12.4929	0.7810	0.0017	13.0903	13.8735
D5	24	76	0.85	2.70	3.41	10.79	1.5370	0.0034	1.2167	2.7613	0.0108	10.7799	10.7893	1.5478	0.0034	11.9966	13.5506
D6	32	68	1.14	2.41	4.54	9.66	2.0493	0.0045	1.6222	3.6818	0.0097	9.6452	9.6536	2.0590	0.0045	11.2674	13.3354
D7	38	62	1.35	2.20	5.40	8.80	2.4336	0.0054	1.9264	4.3721	0.0088	8.7941	8.8018	2.4424	0.0054	10.7205	13.1739
D8	42	57	1.49	2.02	5.99	8.21	2.7026	0.0060	2.1393	4.8323	0.0082	8.1984	8.0920	2.7108	0.0060	10.3377	12.9243
D9	50	50	1.78	1.78	7.10	7.10	3.2021	0.0071	2.5347	5.7528	0.0071	7.0920	7.0982	3.2092	0.0071	9.6267	12.8510
D10	57	42	2.02	1.49	8.21	5.99	3.7016	0.0082	2.9301	6.5582	0.0060	5.9857	5.9625	3.7076	0.0082	8.9158	12.5207
D11	62	38	2.20	1.35	8.80	5.40	3.9706	0.0088	3.1430	7.1334	0.0054	5.3900	5.3947	3.9760	0.0088	8.5330	12.5281
D12	68	32	2.41	1.14	9.66	4.54	4.3549	0.0097	3.4472	7.8238	0.0045	4.5389	4.5429	4.3594	0.0097	7.9861	12.3666
D13	76	24	2.70	0.85	10.79	3.41	4.8672	0.0108	3.8527	8.7442	0.0034	3.4042	3.4071	4.8706	0.0108	7.2569	12.1514
D14	88	12	3.12	0.43	12.50	1.70	5.6357	0.0125	4.4611	10.1249	0.0017	1.7021	1.7036	5.6374	0.0125	6.1632	11.8285
D15	93	7	3.30	0.25	13.21	0.99	5.9559	0.0132	4.7145	10.7002	0.0010	0.9929	0.9938	5.9569	0.0132	5.7074	11.6939
D16	97	3	3.44	0.11	13.77	0.43	6.2121	0.0138	4.9173	11.1604	0.0004	0.4255	0.4259	6.2125	0.0138	5.3428	11.5863
D17	100	0	3.55	0.00	14.20	0.00	6.4042	0.0142	5.0694	11.5056	0.0000	0.0000	0.0000	6.4042	0.0142	5.0694	11.5056
LY-HS	34	66	1.21	2.34	4.90	9.30	2.2099	0.0049	1.7493	3.9119	0.0093	9.2896	9.3697	2.2192	0.0049	11.0389	13.2815
HY-LS	55	45	1.95	1.60	7.80	6.40	3.5178	0.0078	2.7846	6.3281	0.0064	6.3928	6.3884	3.5242	0.0078	9.1774	12.7165

[a] Diets D1-D17 were prepared for Experiment 1, diets Low yeast-high sucrose (LY-HS) and High yeast-low sucrose (HY-LS) were prepared for Experiment 2. In Experiment 1 (diets D1-D17), no lavae survived to adulthood in the diets outside the rows marked in yellow.

[b] Yeast:sucrose mixtures as described in the main text.

[c] This is the weight of yeast:sucrose mixtures added to the whole experimental diet (i.e., 3.55 g of a mixture + 21.5 g of a basic diet as described in the main text).

[d] The proportional contribution of yeast and sucrose to 25 g of whole experimental diet including: 19% corncob fractions, 5.3% corn flour, 0.5% conservatives, 0.44% citric acid, 0.1% guar gum and 60.46% water.

[e] This is the percentage of protein, lipid and carbohydrate, and the kcal content in the portion of yeast and sucrose on 25 g of whole experimental diet.

[f] Expressed as the sum of specific nutrient and kcal input from individual yeast and sucrose. In addition to the nutrients and kcal provided by yeast and sucrose, all diets had 1.10% protein, 0.30% lipid, 5.20% carbohydrate, and 11.006 kcal from corn flour (1.33 g) and corncob fractions (4.75 g) in the basic diet.

Table S2 Two component mixture experimental design layout and recorded response variables.

Run No.	Mixture components (%)		Response variables					
	Dried yeast	Sucrose	Survival to pupa (proportion)	Duration of the larval stage (days)	Pupal weight (mg)	Adult emergence (proportion)	Duration of the pupal stage (days)	Desiccation and starvation resistance (hours)
1	62	38	0					
2	3	97	0					
3	3	97	0					
4	88	12	0					
5	32	68	0.92	11.1	14	1	15.8	78.8
6	24	76	0.92	12.2	15.69	1	16.2	84
7	62	38	0.88	9.9	13.04	1	15.4	84.5
8	57.8	42.2	0.96	9.7	16.15	0.958	15.6	78.8
9	88	12	0.72	9.6	10.98	0.944	15.2	64.2
10	100	0	0					
11	68	32	0.96	9.2	14.97	1	15.1	81
12	76	24	0.88	9.5	14.39	0.955	15.2	81.7
13	32	68	0.8	11.4	13.86	0.95	16.1	78.3
14	93	7	0.16	9.5	6.85	0.5	14	36
15	93	7	0					
16	76	24	0.68	10.6	8.24	0.882	15	65.6
17	0	100	0					
18	100	0	0					
19	7	93	0					
20	0	100	0					
21	7	93	0					
22	0	100	0					
23	50	50	0.88	10.1	14.25	1	15.5	81.8
24	50	50	0.96	9.9	18.05	1	15.4	81.3
25	38	62	1	10.1	18.09	1	15.8	81.6
26	68	32	0.92	9.5	12.97	0.957	15.2	73.5
27	76	24	0.8	9.7	10.25	0.95	15.1	73.5
28	38	62	0.96	10.5	17.65	1	15.9	77.5

Table S2 *Continued.*

Run No.	Mixture components (%)		Response variables					
	Dried yeast	Sucrose	Survival to pupa (proportion)	Duration of the larval stage (days)	Pupal weight (mg)	Adult emergence (proportion)	Duration of the pupal stage (days)	Desiccation and starvation resistance (hours)
29	24	76	0.68	12.6	11.59	1	15.7	72.71
30	57.8	42.2	0.76	10.2	11.49	0.895	15.4	84.7
31	12	88	0					
32	42.2	57.8	0.8	10.6	16.05	1	16	79.2
33	68	32	0.36	9.9	7.17	0.444	15	81
34	62	38	0.92	9.6	13.99	0.957	15.2	80.2
35	97	3	0					
36	12	88	0					
37	3	97	0					
38	38	62	0.84	10.8	14.73	0.952	16.2	86.4
39	7	93	0					
40	32	68	0.36	12.1	8.4	0.777	16	77.1
41	100	0	0					
42	88	12	0.4	9.8	7.84	0.8	14.8	55.5
43	12	88	0					
44	57.8	42.2	0.92	10	16.18	0.957	15.7	90.5
45	50	50	0.92	9.9	15.61	1	15.7	89.2
46	42.2	57.8	1	10.1	16.78	1	16	88.3
47	97	3	0					
48	93	7	0					
49	24	76	0.84	13	12.25	0.955	16	80.6
50	42.2	57.8	0.76	10.9	17.02	1	15.8	92.8
51	97	3	0					
52	0	100	0					
53	7	93	0					
54	3	97	0					
55	32	68	0.8	11.5	13.61	0.95	16.2	84
56	12	88	0					
57	12	88	0					

Table S2 *Continued.*

Run No.	Mixture components (%)		Response variables					
	Dried yeast	Sucrose	Survival to pupa (proportion)	Duration of the larval stage (days)	Pupal weight (mg)	Adult emergence (proportion)	Duration of the pupal stage (days)	Desiccation and starvation resistance (hours)
58	68	32	0.4	10.1	8.25	0.7	15.2	74
59	50	50	0.88	10.9	14.52	0.954	15.6	86.9
60	68	32	0.96	9.7	15.77	1	15.3	84.5
61	50	50	0.28	11.7	5.36	0.429	16	80
62	38	62	0.68	12.3	8.85	0.882	16.1	85.6
63	100	0	0					
64	76	24	0.88	10.1	13.33	1	15.8	77.5
65	3	97	0					
66	76	24	0					
67	88	12	0					
68	7	93	0					
69	97	3	0					
70	24	76	0.56	13.3	6.46	0.642	16.6	78.7
71	97	3	0.52	10.1	6.41	0.538	14.7	42.9
72	93	7	0					
73	38	62	0.64	11.1	11.27	0.938	15.9	90.4
74	93	7	0					
75	32	68	0.8	11.5	13.41	1	16.4	88.8
76	100	0	0					
77	88	12	0					
78	0	100	0					
79	62	38	0					
80	24	76	0					
81	62	38	0.04	16	3.4	0		
82	42.2	57.8	0.96	10.3	19.76	1	16	91.5
83	57.8	42.2	0					
84	57.8	42.2	0.76	10.7	9.75	1	15.5	93.3
85	42.2	57.8	0.16	11.5	4.03	0.25	14	72

Table S3 Two-factor interaction experimental design layout and recorded response variables

Run No.	Chlorogenic acid (mg/100 g artificial diet)	Diet	Response variables					
			Survival to adult (proportion)	Development time to adulthood (days)	Duration of the larval stage (days)	Duration of the pupal stage (days)	Pupal weight (mg)	Desiccation and starvation resistance (hours)
1	16	High yeast-low sucrose	0.44	25.27	9.61	15.44	17.05	90.67
2	0	Low yeast-high sucrose	0.52	25.38	9.79	15.62	19.85	84.00
3	0	High yeast-low sucrose	0.56	25.07	10.14	14.86	20.04	84.00
4	16	Low yeast-high sucrose	0.6	25.27	10.27	14.67	18.28	84.00
5	32	High yeast-low sucrose	0.6	25.13	10.07	15.12	20.03	88.50
6	16	Low yeast-high sucrose	0.56	25.21	10.5	14.5	18.61	108.00
7	0	Low yeast-high sucrose	0.6	26.40	10.93	15.71	19.33	72.00
8	16	High yeast-low sucrose	0.6	24.67	9.6	15.33	20.25	94.00
9	0	High yeast-low sucrose	0.52	24.92	10.15	14.87	19.85	73.50
10	16	High yeast-low sucrose	0.44	25.18	10.23	15.43	17.44	80.57
11	32	High yeast-low sucrose	0.6	25.33	9.8	15.78	20.63	84.00
12	0	High yeast-low sucrose	0.52	24.92	10.23	14.86	19	78.86
13	32	Low yeast-high sucrose	0.6	25.27	11.07	14.29	18.69	80.57
14	16	Low yeast-high sucrose	0.56	24.71	10	14.89	21	85.33
15	16	Low yeast-high sucrose	0.52	26.46	11.46	14.83	16.12	76.00
16	32	High yeast-low sucrose	0.4	24.80	10.69	14	17.28	87.00
17	0	Low yeast-high sucrose	0.56	25.57	10.57	14.75	19.41	93.00
18	32	High yeast-low sucrose	0.52	25.38	9.77	16	19.47	99.43
19	16	High yeast-low sucrose	0.6	25.40	9.6	16	20.5	99.43
20	32	Low yeast-high sucrose	0.48	26.75	12.23	14.62	13.03	85.50
21	0	Low yeast-high sucrose	0.56	26.14	11.21	14.4	18.76	79.20
22	32	Low yeast-high sucrose	0.4	27.60	13.27	14.6	10.69	69.60
